# Fossil Dolphin *Otekaikea marplesi* (Latest Oligocene, New Zealand) Expands the Morphological and Taxonomic Diversity of Oligocene Cetaceans

**DOI:** 10.1371/journal.pone.0107972

**Published:** 2014-09-24

**Authors:** Yoshihiro Tanaka, R. Ewan Fordyce

**Affiliations:** Department of Geology, University of Otago, Dunedin, New Zealand; Penn State University, United States of America

## Abstract

The Oligocene Epoch was a time of major radiation of the Odontoceti (echolocating toothed whales, dolphins). Fossils reveal many odontocete lineages and considerable structural diversity, but whether the clades include some crown taxa or only archaic groups is contentious. The New Zealand fossil dolphin “*Prosqualodon” marplesi* (latest Oligocene, ≥23.9 Ma) is here identified as a crown odontocete that represents a new genus, *Otekaikea*, and adds to the generic diversity of Oligocene odontocetes. *Otekaikea marplesi* is known only from the holotype, which comprises a partial skeleton from the marine Otekaike Limestone of the Waitaki Valley. *Otekaikea marplesi* was about 2.5 m long; it had procumbent anterior teeth, and a broad dished face for the nasofacial muscles implicated in production of echolocation sounds. The prominent condyles and unfused cervical vertebrae suggest a flexible neck. A phylogenetic analysis based on morphological features places *Otekaikea marplesi* in the extinct group Waipatiidae, within the clade Platanistoidea. The phylogeny implies an Oligocene origin for the lineage now represented by the endangered Ganges River dolphin (*Platanista gangetica*), supporting an Oligocene history for the crown Odontoceti.

## Introduction

The history of crown Cetacea, or Neoceti, during the Oligocene Epoch included a dramatic radiation of Odontoceti (dolphins and toothed whales), leading to many taxonomically and morphologically diverse lineages (Whitmore and Sanders [1], Fordyce and Muizon [2], Uhen and Pyenson [3], Fordyce [4]). Morphologically-based phylogenies for the Odontoceti offer two different interpretations of the Oligocene lineages, with wider implications for cetacean evolution: either Oligocene odontocetes are mainly stem taxa, with the crown lineages radiating in the Neogene (e.g., Geisler and Sanders [5], Uhen [6], Geisler et al. [7], Aguirre-Fernandez and Fordyce [8], Geisler et al. [9]); or Oligocene odontocetes include species that represent early members of crown lineages (e.g., Fordyce [10], Murakami et al. [11]). Molecular phylogenetic studies also show the crown Odontoceti radiation starting in the Oligocene (e.g., Steeman et al. [12]), or in the Neogene (e.g., Xiong et al. [13]). Of particular note for this article, the morphologically-based studies show quite variable phylogenetic positions of taxa in the Platanistoidea (*sensu* Muizon [14]), the group that represents the living endangered Ganges and Indus River dolphin, *Platanista gangetica*. The conflicting phylogenies probably reflect limited sampling of Oligocene taxa and morphologies: although many Oligocene odontocetes are known from museum collections, few have been named, few have been described from material other than skulls, and few have reliable stratigraphic dates. The best way to advance knowledge of the early radiation of odontocetes is to describe and interpret fossils that are morphologically and phylogenetically revealing, and geochronologically well-dated, either new species, or poorly-understood named species, as for "*Prosqualodon*" *marplesi* reported here. Such activity should produce better-established and better-dated clades, with implications for cetacean and mammalian molecular clocks and for understanding of physical versus biological drivers of cetacean evolution and extinction [15], [16].

New Zealand is one of few regions in the world with widespread, well-dated, fossiliferous Oligocene rocks that have produced well-preserved fossil Cetacea (Fordyce [4], [17]). One species from the Waitaki Valley of New Zealand, *Prosqualodon marplesi* Dickson 1964 (Otekaike Limestone, latest Oligocene), is detailed here. Dickson [18] briefly described the holotype and unique specimen, and identified it as representing a new species in the austral genus *Prosqualodon* because “the nasals overhang the nostrils, a feature of the prosqualodonts” (page 627). More preparation of the holotype (by the authors, and by preparators A. Grebneff and S.E. White) revealed many fine details, and led to the discovery of the taxonomically important periotic. The aim here is to describe the morphology, consider function, and assess the phylogenetic relationships. The redescription will help assess the suggestion (Fordyce, [10]) that *P. marplesi* might be an early species in the lineage of Platanistoidea, leading to the living endangered Ganges and Indus River dolphin, *Platanista gangetica*.

Fordyce [10] recognised *P. marplesi* as a member of the genus *Notocetus* (Squalodelphinidae), rather than the genus *Prosqualodon* Lydekker 1894, and suggested the new combination *Notocetus marplesi* (Dickson, 1964). Hitherto, the genus *Notocetus* was known only from one species, the early Miocene *Notocetus vanbenedeni* from the Atlantic margin of South America ([14], [19], [20]). Concurrent with proposing the combination *Notocetus marplesi*, Fordyce [10] named a new species, genus and family, *Waipatia maerewhenua* Fordyce, 1994 (Waipatiidae) for a unique late Oligocene dolphin from Waipati, Waitaki Valley, South Island, New Zealand. Fordyce [10] mentioned other small odontocetes that might be Waipatiidae, including *Sachalinocetus cholmicus* Dubrovo in Siryk and Dubrovo, 1970 (early Miocene, Russia), and *Sulakocetus dagestanicus* Mchedlidze, 1976 (latest Oligocene, Georgia). Later, Bianucci [21] noted late early Miocene specimens of possible Waipatiidae from Malta. We have not been able to directly examine the putative waipatiids from beyond New Zealand, there are too few details published to allow cladistic coding and phylogenetic analysis, and for now we place them incertae sedis. The early Miocene archaic odontocete from New Zealand, *Papahu taitapu*, Aguirre-Fernandez and Fordyce, 2014 was placed by Aguirre-Fernandez and Fordyce next to, but not in, the clade for *W. maerewhenua* [8], in a basal to crownward sequence as follows: (basal) [*Simocetus*+*Agorophius*] - *Waipatia* - *Papahu* - *Prosqualodon* - *Zarhachis -* [*Notocetus*+*Squalodon*] - *Xiphiacetus* - crown Odontoceti. Previous hypotheses on the relationships of *P. marplesi* and supposed relatives are considered further below.

## Materials, Methods, Approvals

### Acronyms

**AMNH** - American Museum of Natural History, New York, U. S. A.; **MLP**- Museo de La Plata, La Plata, Argentina, **NSM**- National Museum of Nature and Science, Tskuba, Japan; **OM -** Otago Museum, Dunedin, New Zealand (**OM C** and **OM GL** are historic and current catalogs, respectively, in OM); **OU** - Geology Museum, University of Otago, Dunedin, New Zealand; **USNM** - Department of Paleobiology, National Museum of Natural History, Smithsonian Institution, Washington, D. C., U. S. A. All these institutions are accessible, permanent repositories.

### Methods

Material was prepared using pneumatic chisels and hand tools, with finishing carried out under a Zeiss SR binocular microscope at 8 or 12 x. The positions of most sutures were confirmed using the binocular microscope. Photographs were taken with a Nikon D700 DSLR camera and a 105 mm micro lens. Most views show the specimen coated with sublimed ammonium chloride, with lighting from the upper left.

### Ethics statement

No permits were required for the described study, which complied with all relevant regulations.

### Nomenclatural Acts

The electronic edition of this article conforms to the requirements of the amended International Code of Zoological Nomenclature, and hence the new names contained herein are available under that Code from the electronic edition of this article. This published work and the nomenclatural acts it contains have been registered in ZooBank, the online registration system for the ICZN. The ZooBank LSIDs (Life Science Identifiers) can be resolved and the associated information viewed through any standard web browser by appending the LSID to the prefix "http://zoobank.org/". The LSID for this publication is: urn:lsid:zoobank.org:pub: 3F8320E1-9402-4E29-835A-8BFB0E1904E9. The electronic edition of this work was published in a journal with an ISSN, and has been archived and is available from the following digital repositories: PubMed Central, LOCKSS.

### Geological setting

**Figure 1:**
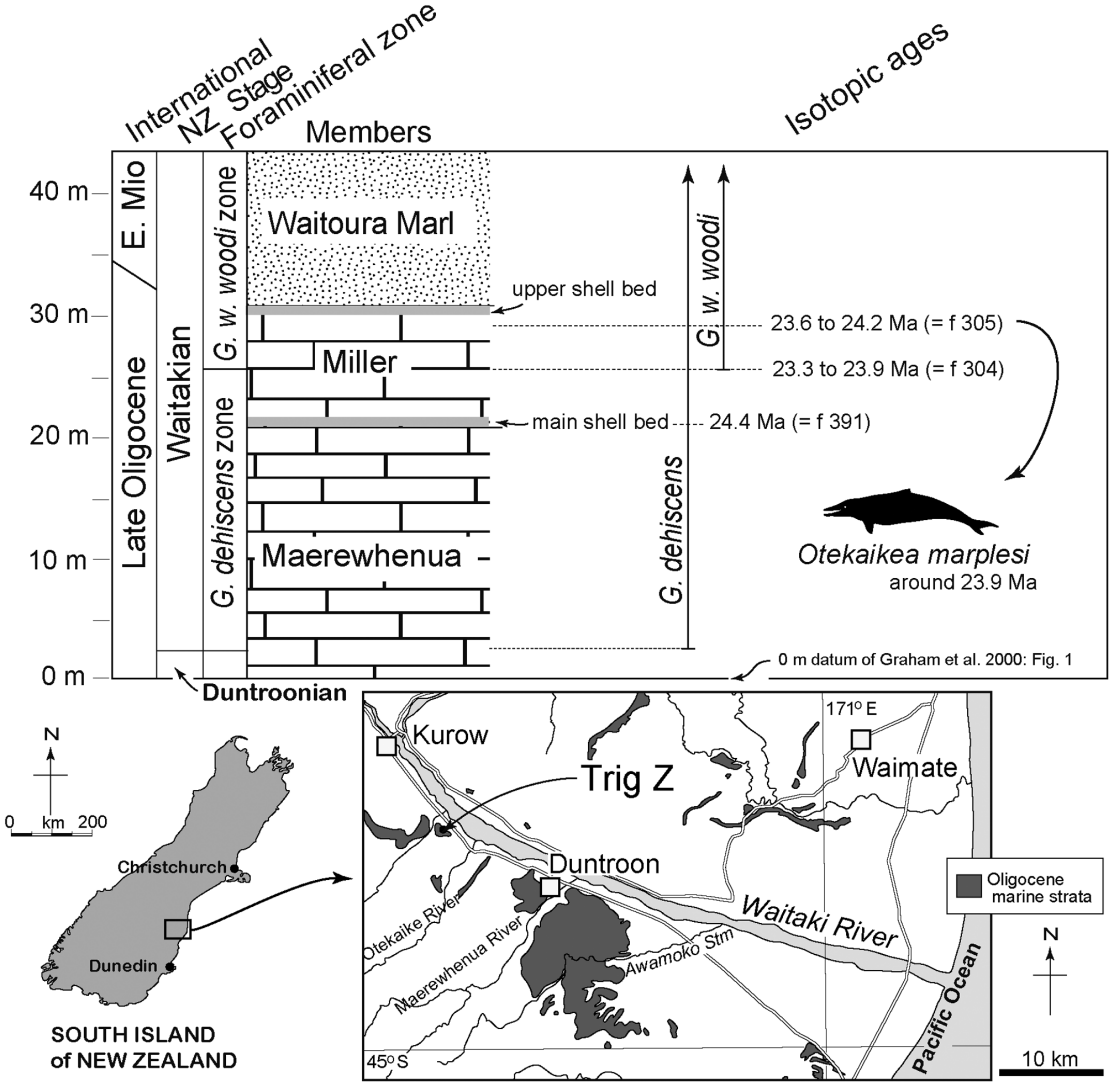
Locality map and stratigraphic sections for the *Otekaikea marplesi*, type locality. Dot shows the type locality of Trig Z, Gards Road, for *Otekaikea marplesi*. The foraminiferal zone and isotopic age are explained in the text. The fossil record numbers (f304, 305, 391) are from Graham et al. [24].

The holotype specimen of *Otekaikea marplesi* was collected from the Otekaike Limestone at Trig Z, Gards Road, Otiake, Waitaki Valley, 13 km west northwest of Duntroon, North Otago (Fig. 1), by T. G. Marples in 1954. The fossil record number is I40/f30 (New Zealand fossil record file, Geological Society of New Zealand). Metric grid reference is NZMS 260 140: 146975; Dickson [18] cited imperial (one inch to one mile) grid reference NZMS 1 S127: 160015 (See also Gage [22]: Geological Map No. 2). Latitude 44°46′49″ S, longitude 170°29′27″ E. The type-locality lies at or near the right side of Gage's ([22]: Fig. 25) photograph of the type locality of the Waitakian Stage. The locality has been prospected, but no other significant material of *O. marplesi* has been found.

The sequence at Trig Z includes the type section for the local Waitakian Stage. There are three successive members of Otekaike Limestone: the basal Maerewhenua Member (soft to cemented massive, glauconitic limestone); the Miller Member (yellow or brown calcareous silt with shellbeds and large low-angle cross-beds); the uppermost Waitoura Marl (massive calcareous silt) [22]–[24]. The top of the Maerewhenua Member is marked by a conspicuous, richly fossiliferous, glauconitic shellbed, the "main" or "lower" shellbed of Jenkins [25]; a second, indifferently exposed "upper" shellbed occurs at about the top of the Miller Member [25]. Dickson [18] reported that the type specimen was taken from “a calcareous shell bed overlying the Otekaike Limestone.” The matrix of the type specimen is not obviously a shell bed, but is light yellow-green, slightly cemented, glauconitic limestone with abundant foraminifera and ostracoda, and bioclasts of fragmented larger invertebrates. The matrix produced rare small specimens of the planktic foraminiferan *Globoturborotalita woodi*, indicating the *G. woodi* planktic foraminiferal zone of Jenkins [26], in the middle of the New Zealand Waitakian stage. No specimens were found of the next younger zonal species *Globoturborotalita connecta*. Previously, the “*Globigerina” woodi* zone was identified as present at Trig Z from the upper part of the Miller Member (middle Waitakian) upwards [24], [25]. The Waitoura Member can be dismissed as the type-horizon because its lithology is massive calcareous siltstone (probably the basal Mount Harris Formation of Beu et al. [27], as interpreted by Fordyce). Both the matrix and planktic foraminifera suggest that *O. marplesi* is from the upper part of the Miller Member of the Otekaike Limestone. *Globigerina brazieri* was also present, indicating an age no younger than Waitakian Stage [25].

Graham et al. [24] gave strontium dates for the Trig Z sequence (derived from analysis of foraminifera and macrofossils, mainly pectinids), including 23.6 to 24.2 Ma for the upper part of the Miller Member (I40/f305; ^87^Sr/^86^Sr values include 0.708263+/−0.000011), and 23.3 to 23.9 Ma for the base of the *G. woodi* zone (I40/f304; ^87^Sr/^86^Sr  = 0.708258+/−0.000011). The age for *Otekaikea marplesi* is taken here as ≥23.9 Ma.

### Systematic paleontology

Cetacea Brisson, 1762

Neoceti Fordyce and Muizon, 2001

Odontoceti Flower, 1867

Platanistoidea Simpson, 1945, *sensu* Muizon, 1987

Waipatiidae Fordyce, 1994

Genus *Otekaikea*, new genus

urn:lsid:zoobank.org:act: 702DACF7-1BB7-4E4E-A851-7EAE20EF5F25

**Type species.** *Prosqualodon marplesi* Dickson 1964.

**Included species.** *Prosqualodon marplesi* Dickson 1964, only.

**Diagnosis.** As for the only included species, *Otekaikea marplesi*, below.

**Comments.** Originally, *Otekaikea marplesi* was named as *Prosqualodon marplesi* by Dickson [18]. Fordyce [10] later used the binomen *Notocetus marplesi*.

**Etymology.** From the Maori name Otekaike, derived from the source formation, Otekaike Limestone (of Gage [22]). Alternatively, Otekaike may be spelled Otekaieke. Pronunciation: o-te-kai-ke, with *o*, the pronounced as in English “oar”, and *ke* as in “chemical”.

*Otekaikea marplesi*

Fig. 2–17 and Table 1–3.

**Figure 2:**
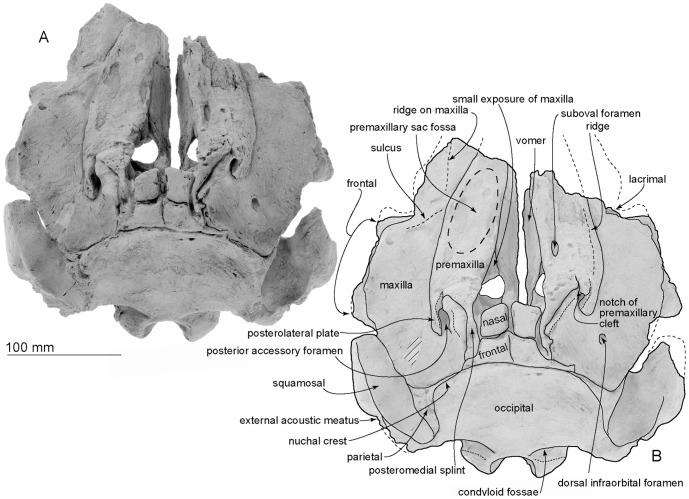
Dorsal views of the type skull, OM GL 421, *Otekaikea marplesi*. Note the asymmetrical vertex, involving the posterior accessory foramina, premaxillary splints, and nodular nasals and frontals. This and other photographs show the fossil whitened by a coating of sublimed ammonium chloride, unless stated otherwise.

**Figure 3:**
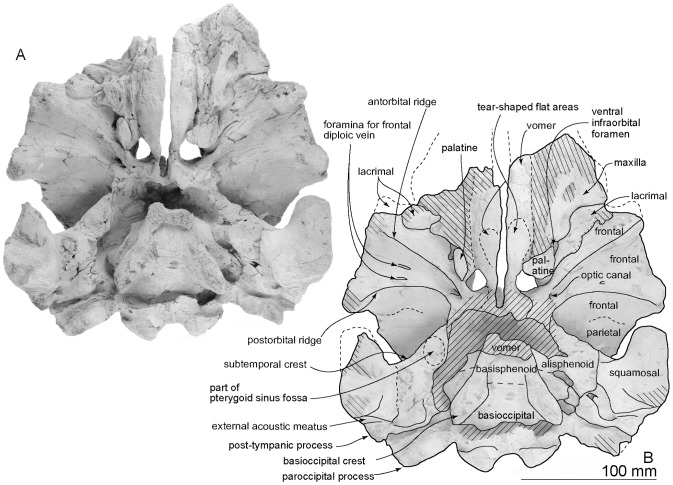
Ventral views of the type skull, OM GL 421, *Otekaikea marplesi*. A more detailed posterior basicranial illustration is in figure 8. Note that the floor of the braincase is crushed dorsally upwards.

**Figure 4:**
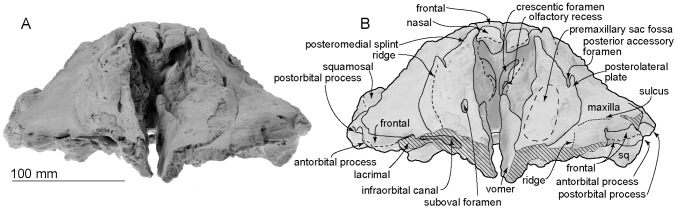
Anterior views of the type skull, OM GL 421, *Otekaikea marplesi*.

**Figure 5:**
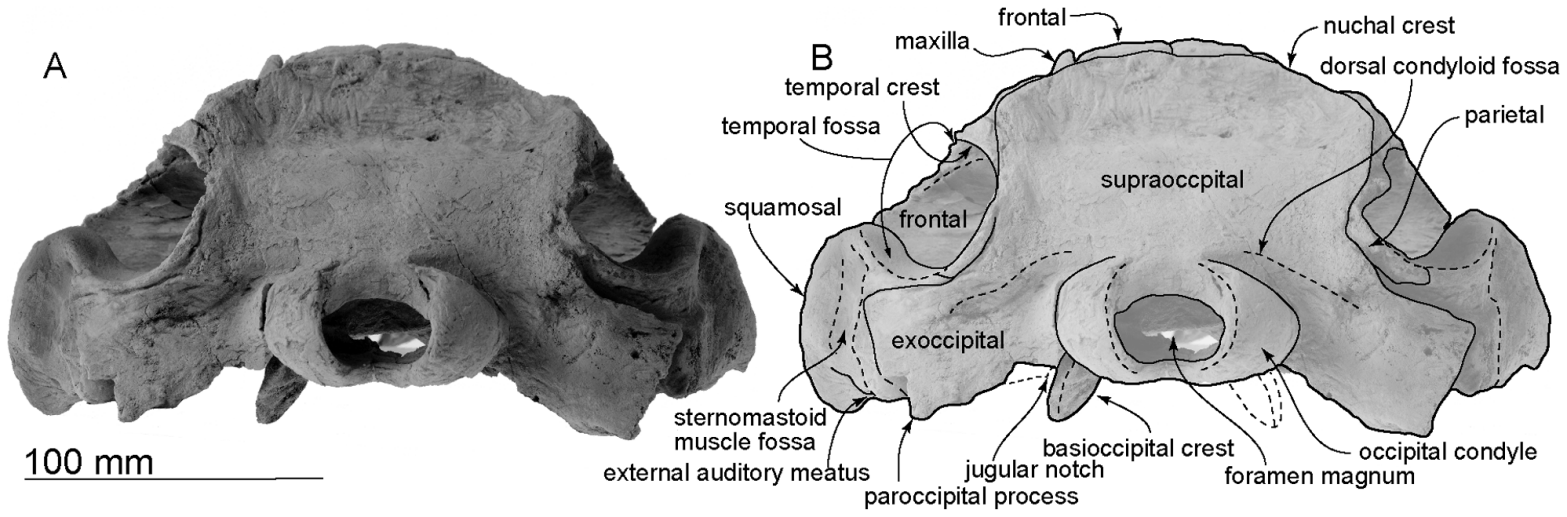
Posterior views of the type skull, OM GL 421, *Otekaikea marplesi*.

**Figure 6:**
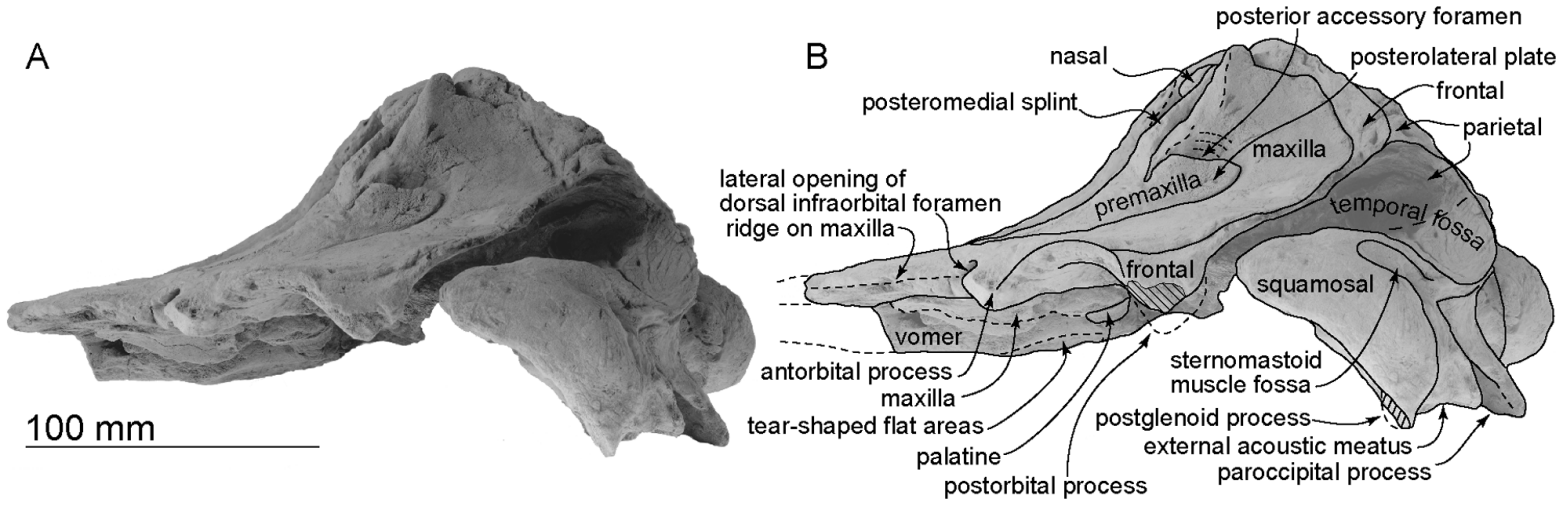
Left lateral views of the type skull, OM GL 421, *Otekaikea marplesi*.

**Figure 7:**
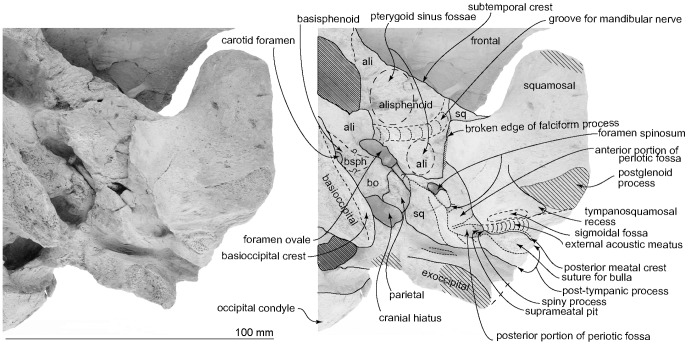
Details of left basicranium of the type skull, OM GL 421, *Otekaikea marplesi*. Note: the foramen spinosum (covered posteriorly by a small tubercle); the large periotic fossa; and the large carotid foramen.

**Figure 8:**
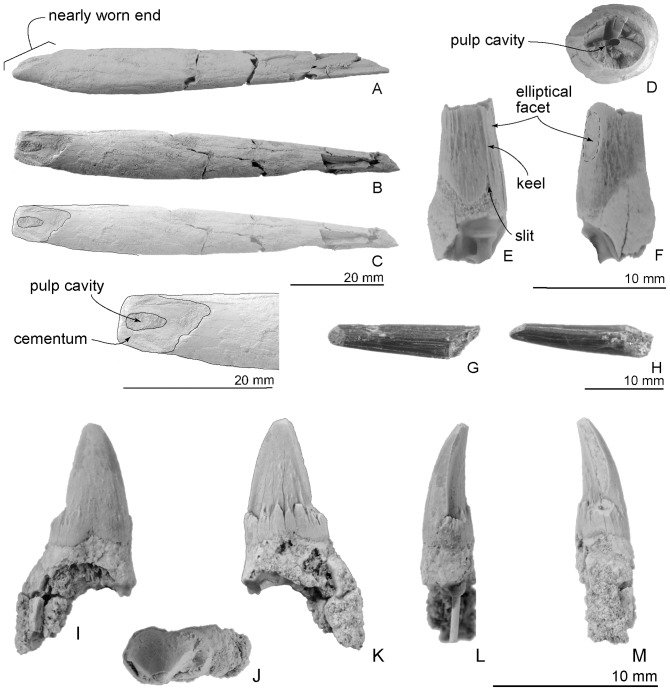
Type teeth, OM GL 421, *Otekaikea marplesi*. Scales vary as indicated, depending on the tooth. A–C, incisor. A, presumed lateral view. B, worn surface view (dorsal or ventral). C, line art of the worn end. D–F, single rooted tooth. D, occlusal view. E, posterior view. F, presumed lateral view. G and H, single rooted tooth. G, presumed dorsal or ventral view. H, presumed lateral view. I–M, double rooted tooth. I occlusal view. J, buccal view. K, lingual view. L, anterior view. M, posterior view. The large tusk (A–C) has an exposed pulp cavity.

**Figure 9:**
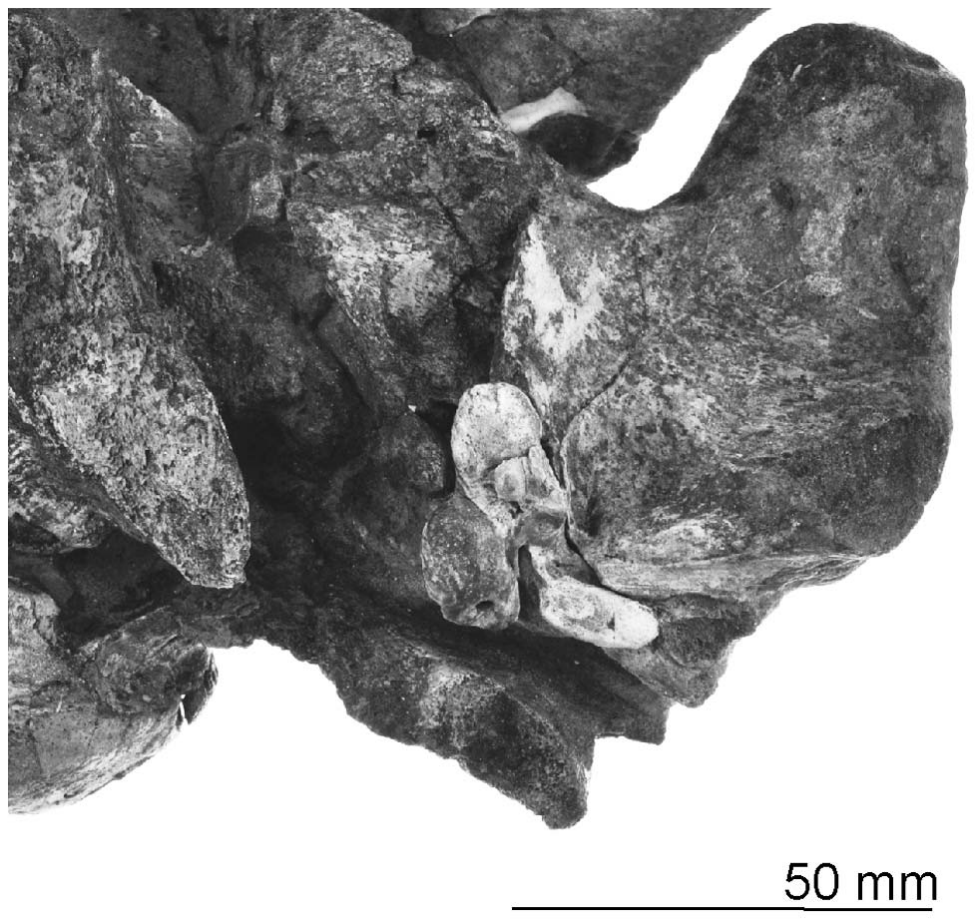
Periotic in original position next to the left squamosal, OM GL 421, *Otekaikea marplesi*. Note that the lateral margin of the articulated closely follows the thin falciform process of the squamosal. Fossil is not coated with sublimed ammonium chloride.

**Figure 10:**
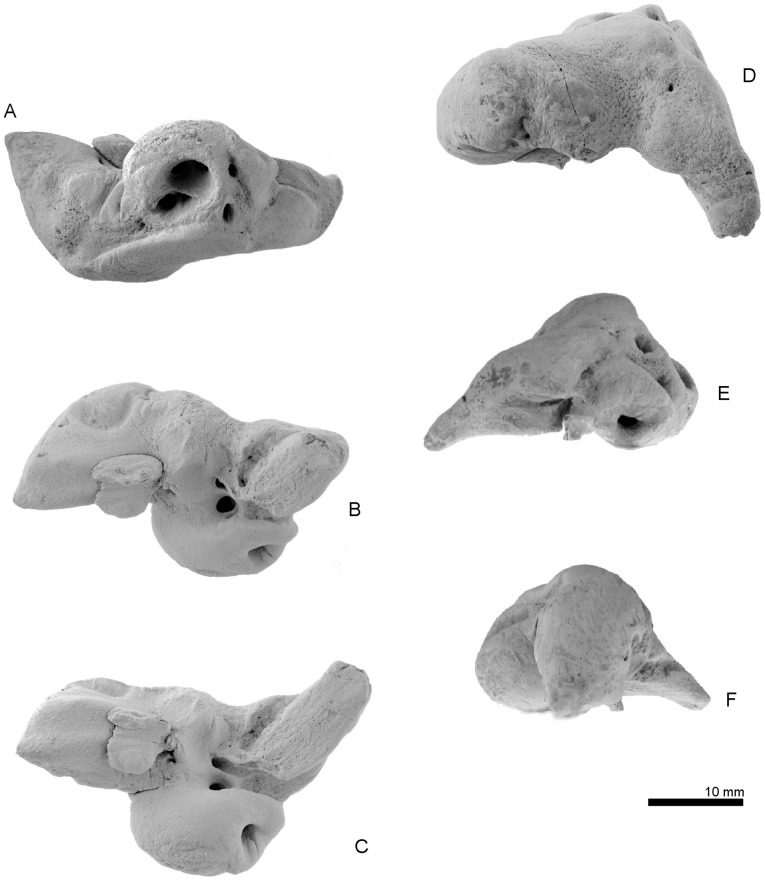
Type left periotic, OM GL 421, *Otekaikea marplesi*. A, medial view. B, lateral view. C, ventral view. D, dorsal view. E, posterior view. F, anterior view.

**Figure 11:**
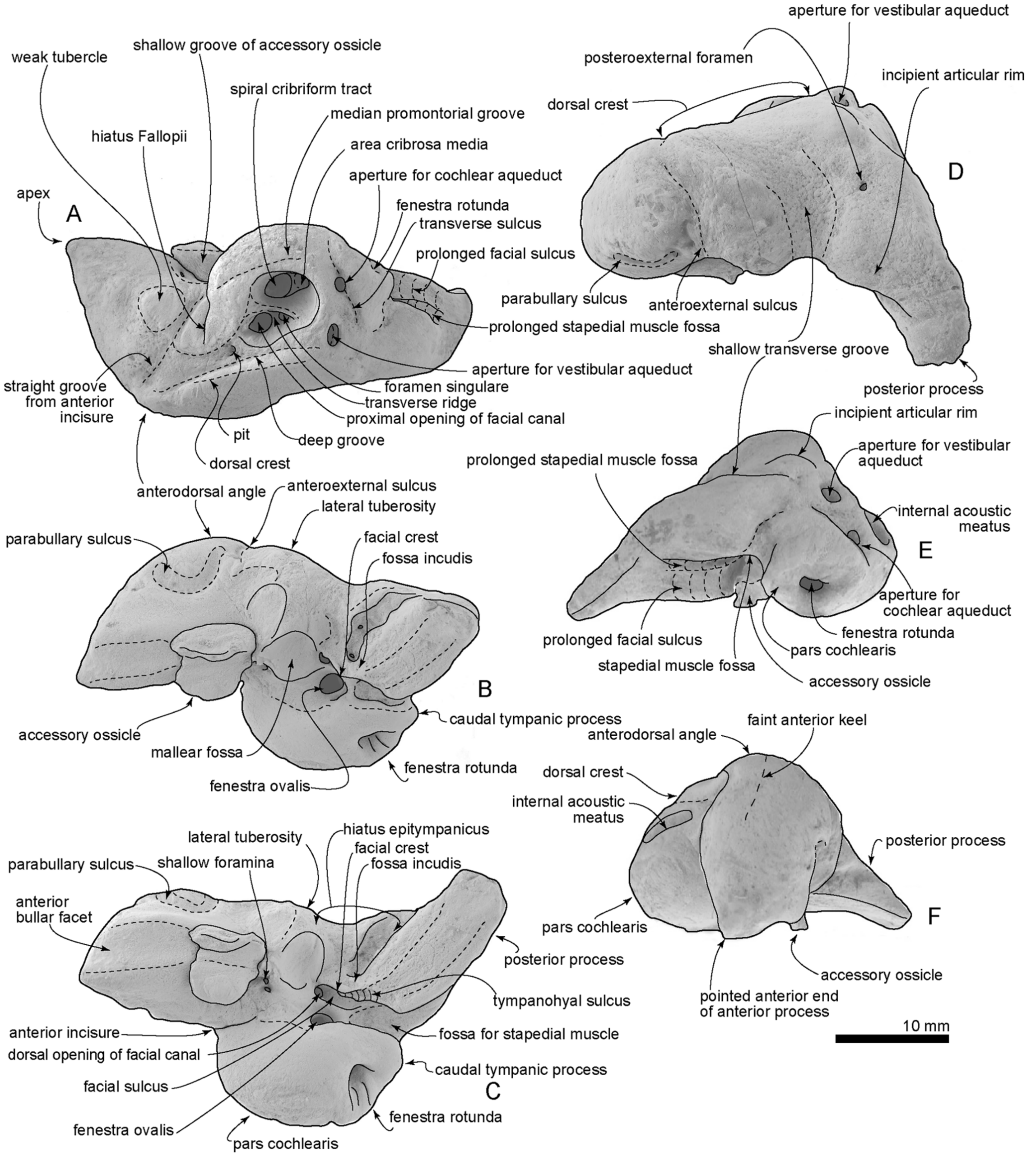
Key features of the type left periotic, OM GL 421, *Otekaikea marplesi*. A, medial view. B, lateral view. C, ventral view. D, dorsal view. E, posterior view. F, anterior view. Note the pointed apex of the anterior process, which is unlike *Waipatia maerewhenua*.

**Figure 12:**
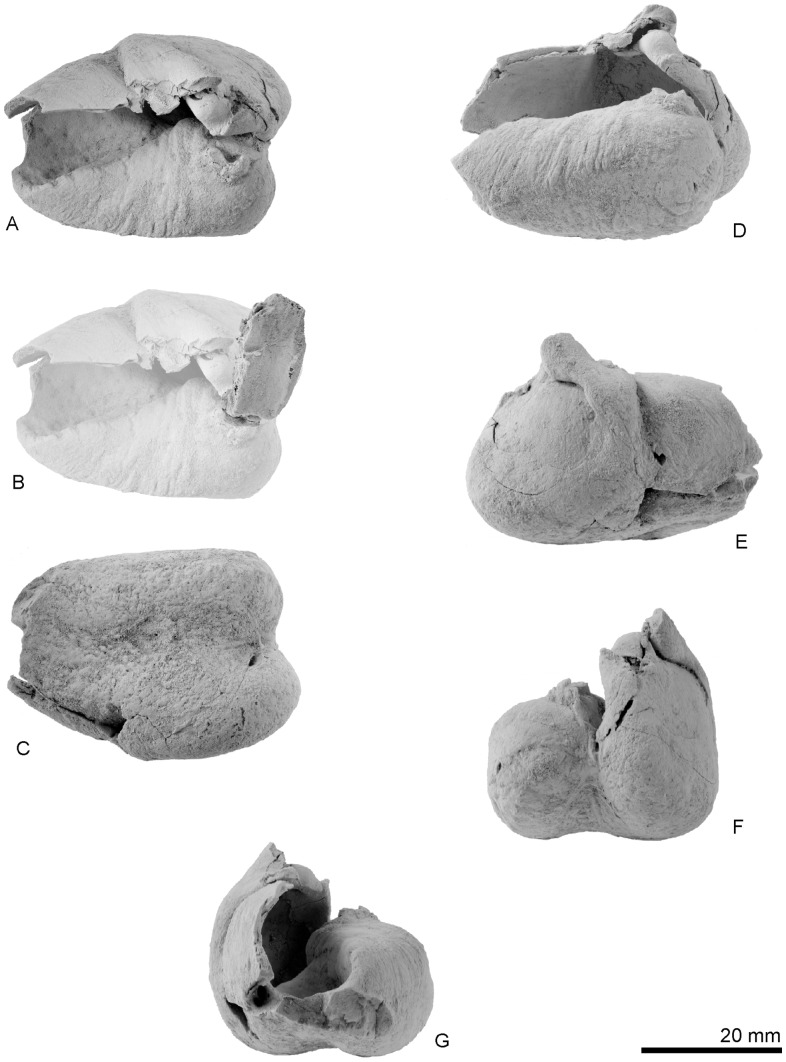
Type right bulla, OM GL 421, *Otekaikea marplesi*. A, dorsal view. B, posterior process, dorsal view. C, ventral view. D, medial view, E, lateral view, F, posterior view, G, anterior view.

**Figure 13:**
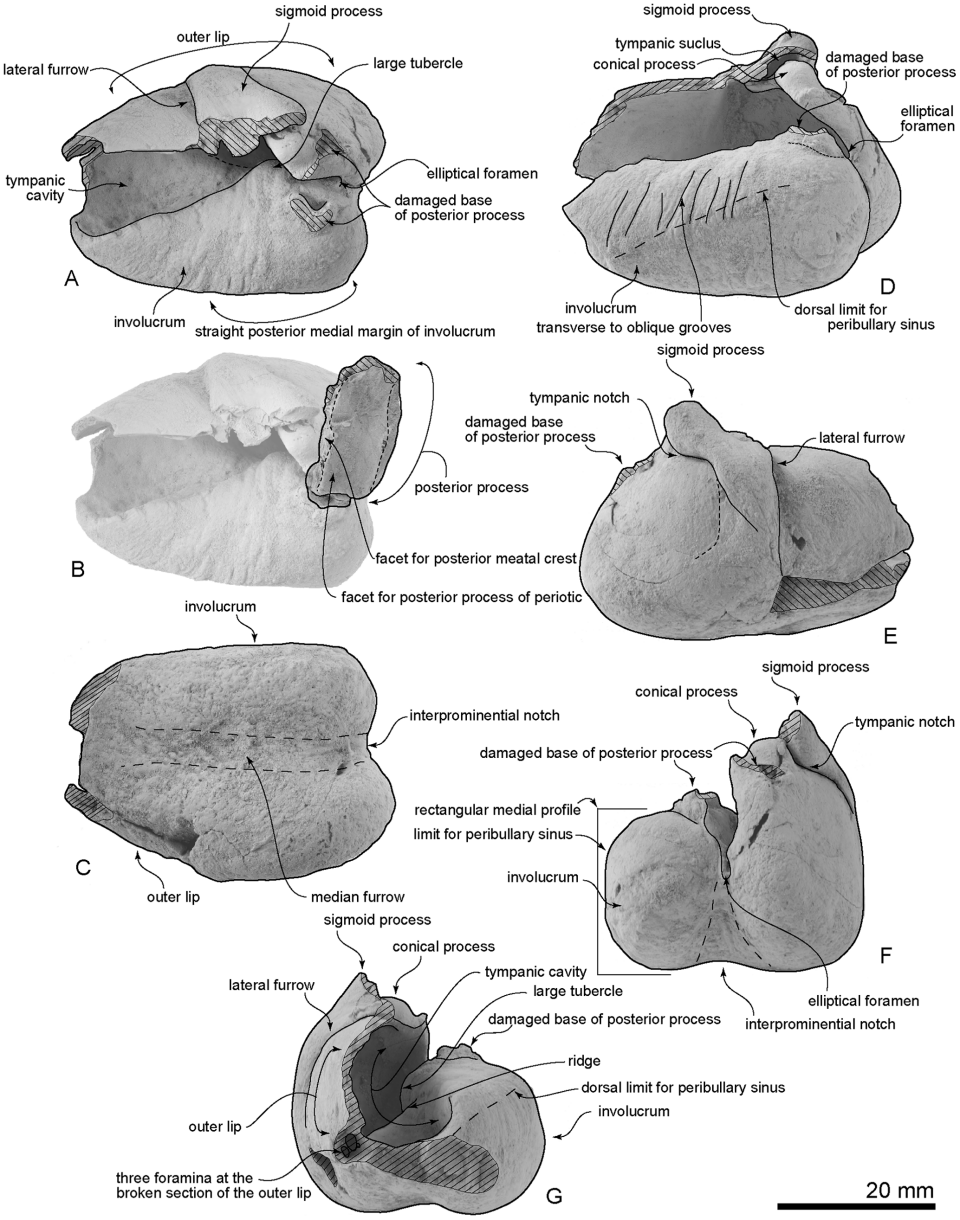
Key features of the type right bulla, OM GL 421, *Otekaikea marplesi*. A, dorsal view. B, posterior process superimposed on figure 13A in proposed life position, dorsal view. C, ventral view. D, medial view. E, lateral view. F, posterior view. G, anterior view.

**Figure 14:**
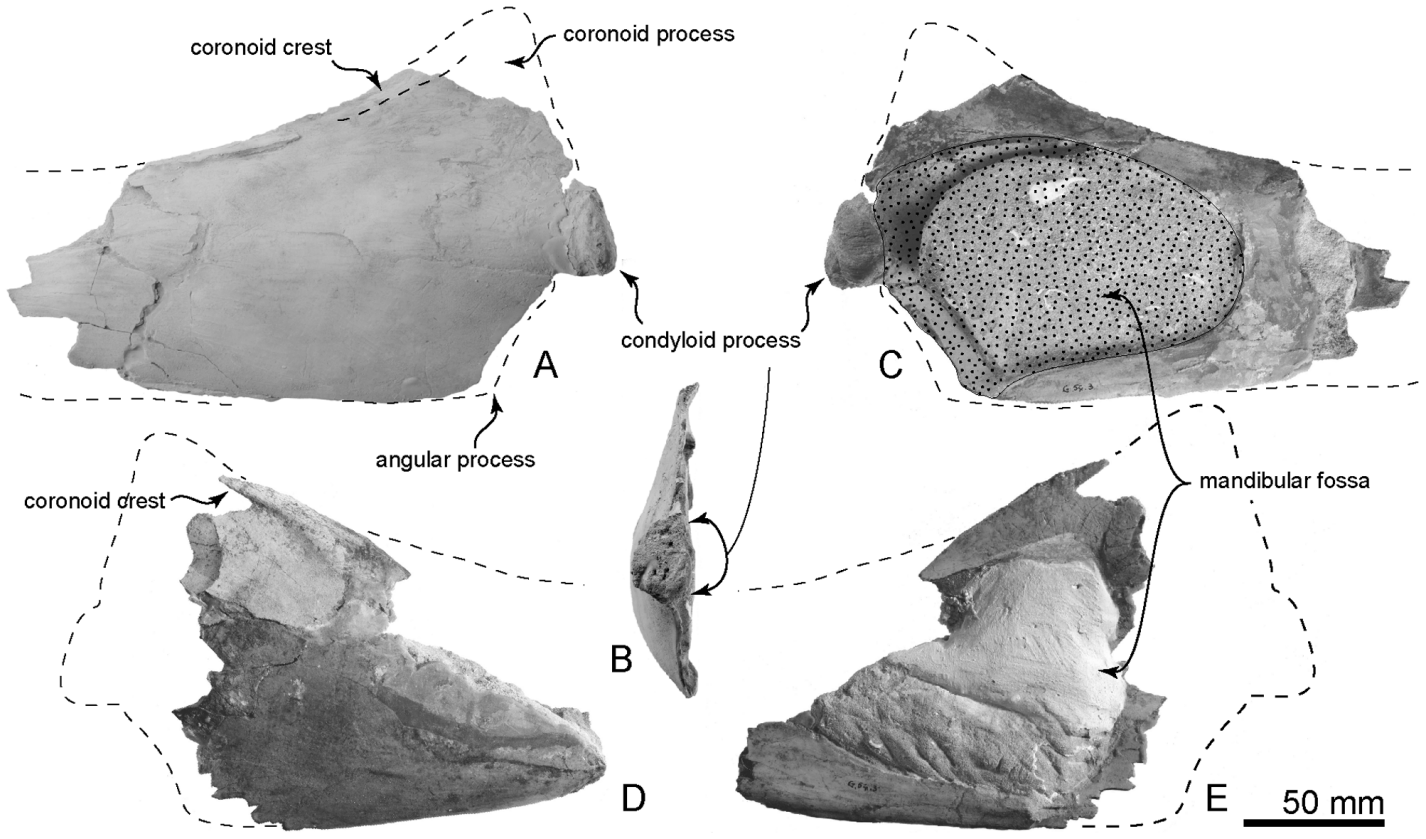
Type mandible, OM GL 421, *Otekaikea marplesi*. A–C, right mandible. A, lateral view. B, posterior view. C, medial view. D and E, left mandible. D, lateral view. E, medial view. Note that the distance between the coronoid process and the condyle is short.

**Figure 15:**
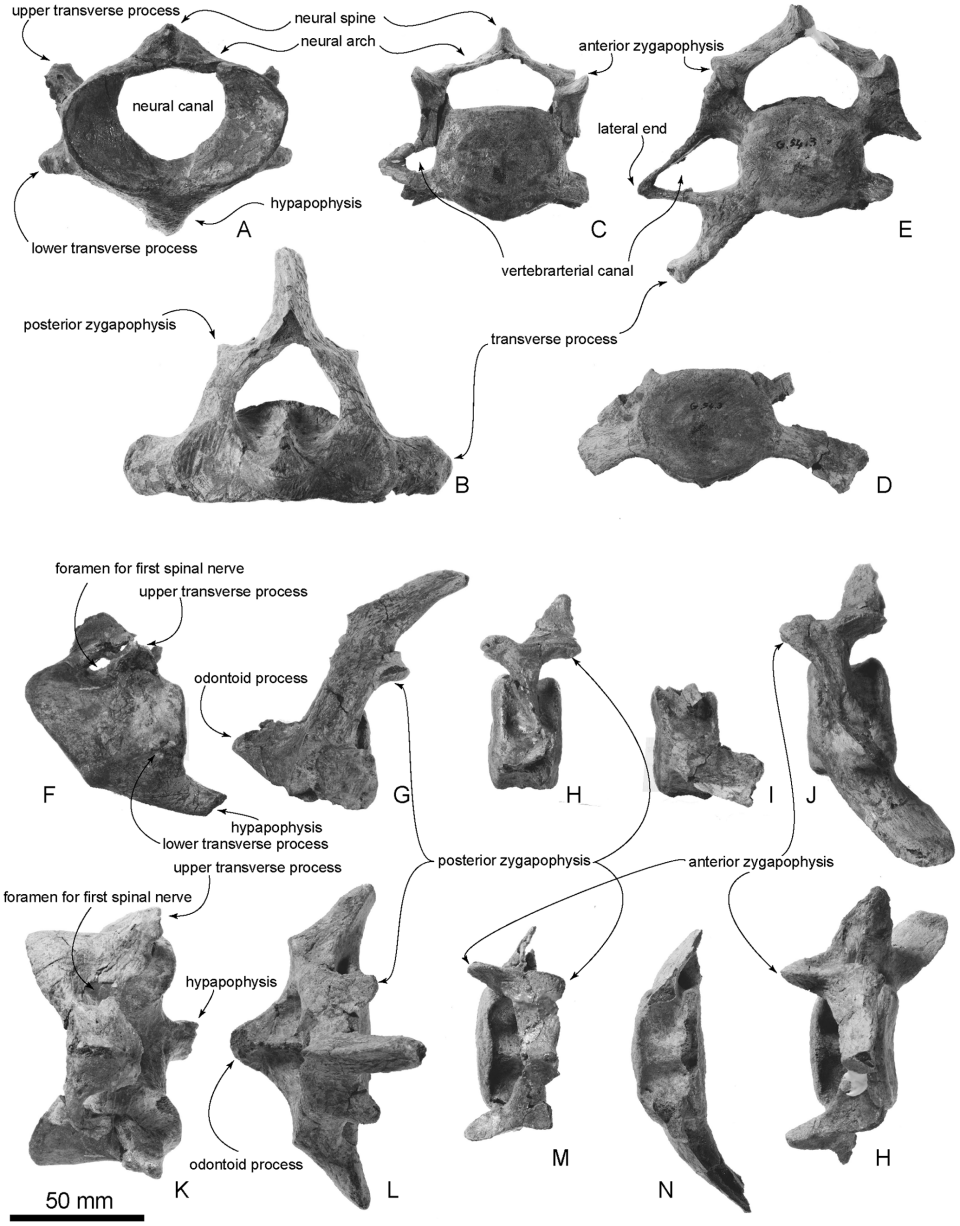
Type cervical vertebrae, OM GL 421, *Otekaikea marplesi*. A–E, anterior views, F–J, lateral views, K–H, dorsal views. A, F and K, atlas. B, G and L, axis. C, G and L, fourth cervical vertebra. I and N, sixth cervical vertebra. E, J and H, seventh cervical vertebra. Note that the cervical vertebrae are relatively long anteroposteriorly and are not fused.

**Figure 16:**
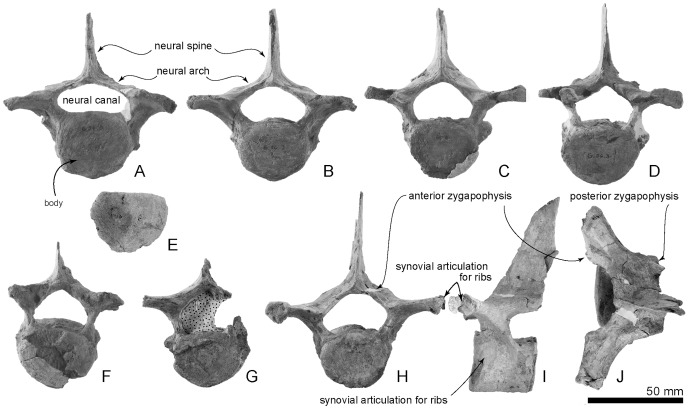
Type thoracic vertebrae, OM GL 421, *Otekaikea marplesi*. A, first thoracic vertebra. B second thoracic vertebra. C, third thoracic vertebra. D, a posterior thoracic vertebra. E, isolated epiphysis. F and G, three posterior thoracic vertebrae. H–J, a posterior thoracic vertebra, anterior, left lateral and dorsal views.

**Figure 17:**
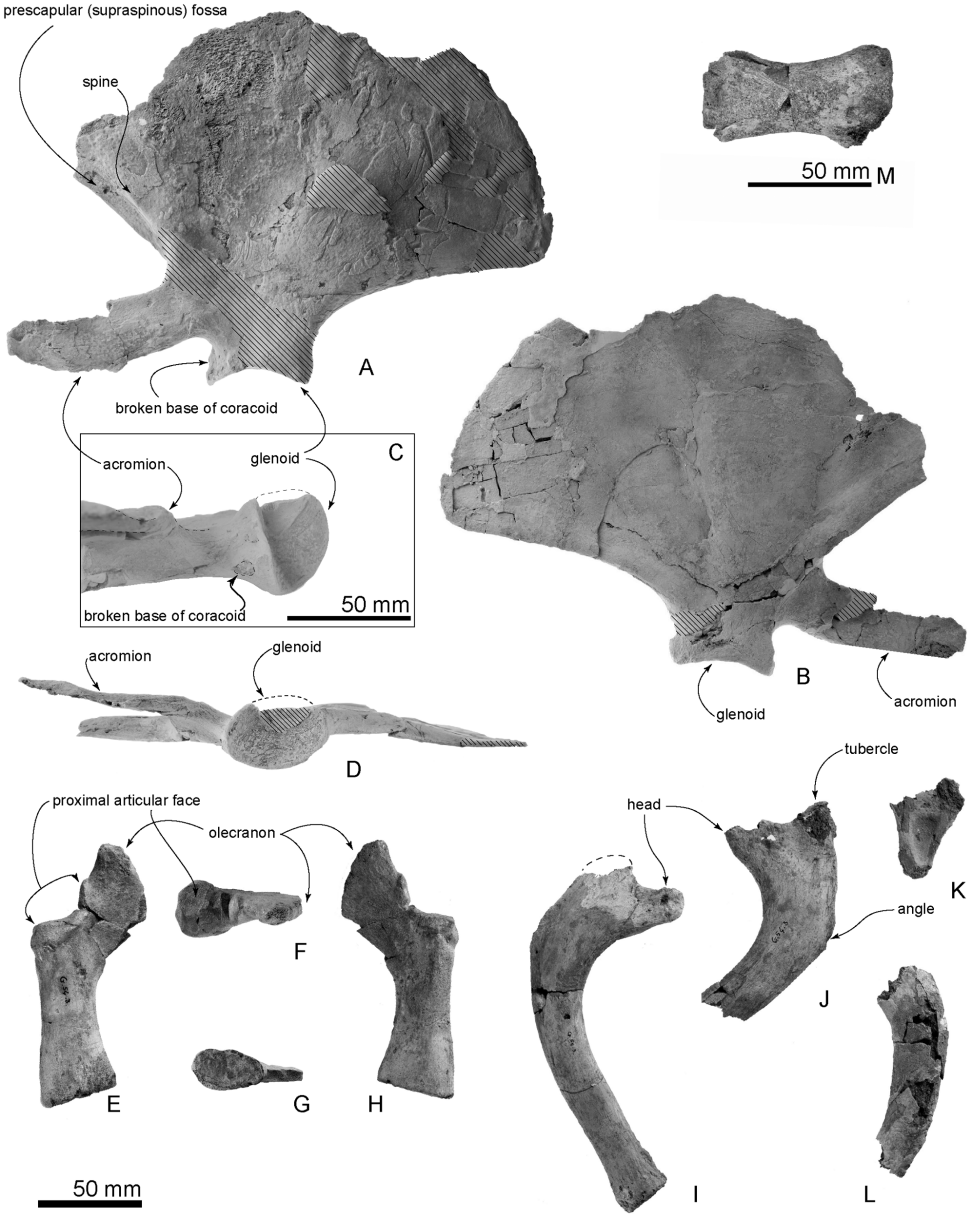
Type forelimb elements and ribs, OM GL 421, *Otekaikea marplesi*. A–D, left scapula. A, lateral view. B, medial view. C, details of glenoid region. D, distal view. E–H, ulna. E, lateral view. F, proximal view. G, distal view. H, medial view. I–L, four ribs. Note that the rounded broken surface on the scapula, C, suggests the presence of a small coracoid process.

**Table 1:** Measurements in mm of holotype, OM GL 421, *Otekaikea marplesi*: skull and mandibles.

**Skull**	
total length from the most anterior point to posterior of occipital condyles	254.0+
cranial length	197.0
width of premaxillae at a line across posterior limits of antorbital notches	40.0
maximum width of premaxillae about the level with mid-orbit	125.0
preorbital width at level of frontal-lacrimal suture	212.0+
postorbital width across apices of postorbital processes	251.0+
distance from broken tip of rostrum to anterior of left bony naris	149.0+
maximum width across narial aperture	51.0
median length of nasals on vertex point to point	23.0
maximum length of right nasal on vertex	28.0
median length of frontals on vertex	19.0
distance from posterior of occipital condyle to anterior apex of supraoccipital	98.5
vertical external height of skull from most ventral part of braincase on basioccipital crest to dorsal extremity of frontal at vertex	120.0+
bizygomatic width	257.0
**Mandibles**	
maximum preserved length of left mandibles	30.5+
maximum preserved height of left mandible	117.0+
height of condyloid process	32.5
width of condyloid process	18.5

Dimensions follow Fordyce et al. [82] and Perrin [33]. Measurements are rounded to the nearest 0.5 mm. For skull and mandible, distances are either horizontal or vertical, unless identified as point to point.

**Table 2:** Measurements in mm of holotype, OM GL 421, *Otekaikea marplesi*: periotic and tympanic bulla.

maximum anteroposterior length from anterior apex of anterior process to apex of posterior process	39.0
maximum anteroposterior length parallel to dorsal margin	36.0
maximum dorsoventral depth anterior process perpendicular to axis of periotic	19.0
length of anterior process from anterior apex to level of posterior of mallear fossa	22.5
length of anterior process from anterior apex of anterior process to level of anterior of pars cochlearis in notch immediately lateral to fine ridge	15.0
dorsoventral depth at fovea epitubaria	12.5
length facet on posterior process point to point	15.0
facial canal anteroposterior diameter	1.5
maximum width of anterior process at base	12.0
approximate anteroposterior length of pars cochlearis	17.0
approximate transverse width of pars cochlearis from internal edge to fenestra ovalis	8.0
transverse width of periotic internal face of pars cochlearis to apex of lateral tuberosity	20.0
length of posterior process of periotic	13.0
length of posterior process parallel to posterior profile/steeply acute to long axis of body	15.5
**Tympanic bulla**	
standard length anterior apex to apex of outer posterior prominence	41.5+
length anterior apex to apex of inner posterior prominence	40.0+
distance outer posterior prominence to apex of conical process	41.0+
approximate width across inner and outer posterior prominences	17.5
dorsoventral depth of involucrum immediately in front of posterior pedicle	18.5
approximate point-point length of posterior process	18.5+

Dimensions follow Fordyce et al. [82], Perrin [33] and Kasuya [83].

Measurements are rounded to the nearest 0.5 mm. For skull and mandible, distances are either horizontal or vertical, unless identified as point to point.

**Table 3:** Measurements in mm of holotype, OM GL 421, *Otekaikea marplesi*: postcranial elements.

maximum preserved length	76.5
maximum preserved height	82.0
maximum preserved width	98.5
length of neural spine	26.5+
height of neural spine	18.5+
length of body	53.0
height of anterior articular surface	44.5
width of anterior articular surface	82.0
height of posterior articular surface	37.0
width of posterior articular surface	78.5
**Axis**	
maximum preserved length	85.5+
maximum preserved height	102.0+
maximum preserved width	124.0
length of neural spine	43.5+
height of neural spine	35.0+
length of body	51.5
length of dens	20.0
height of anterior articular surface	31.5
width of anterior articular surface	78.5
height of posterior articular surface	38.5
width of posterior articular surface	54.0
**4^th^ cervical vertebra**	
maximum preserved length	35.5
maximum preserved height	71.5+
maximum preserved width	79.0+
length of neural spine	14.0+
height of neural spine	11.5+
length of body	25.0
height of anterior articular surface	42.0
width of anterior articular surface	47.0
height of posterior articular surface	40.5
width of posterior articular surface	48.0
**6^th^ cervical vertebra**	
maximum preserved length	32.5+
maximum preserved height	43.5+
maximum preserved width	109.0+
length of body	25.0
height of anterior articular surface	40.5
width of anterior articular surface	50.0
height of posterior articular surface	39.5+
width of posterior articular surface	51.5
**7^th^ cervical vertebra**	
maximum preserved length	56.0
maximum preserved height	108.0+
maximum preserved width	104.0+
length of neural spine	12.5
height of neural spine	10.8+
length of body	29.0
height of anterior articular surface	41.0
width of anterior articular surface	43.5
height of posterior articular surface	43.5
width of posterior articular surface	46.5
**Thoracic vertebra A in ** **Fig. 16** **.**	
maximum preserved length	50.0+
maximum preserved height	109.0+
maximum preserved width	110.0+
length of neural spine	20.0
height of neural spine	48.0
length of body	33.5
**Thoracic vertebra B in ** **Fig. 16** **.**	
maximum preserved length	51.0+
maximum preserved height	116.0+
maximum preserved width	113.0+
length of neural spine	28.5
height of neural spine	50.0
length of body	40.0
**Thoracic vertebra C in ** **Fig. 16** **.**	
maximum preserved length	54.5+
maximum preserved height	116.0+
maximum preserved width	109.0+
length of neural spine	39.0
height of neural spine	49.5+
length of body	42.0
**Thoracic vertebra D in ** **Fig. 16** **.**	
maximum preserved length	61.0+
maximum preserved height	122.0+
maximum preserved width	95.0+
length of neural spine	44.0+
height of neural spine	55.0+
length of body	48.0
**Thoracic vertebra E in ** **Fig. 16** **.**	
maximum preserved length	51.0+
maximum preserved height	94.5+
maximum preserved width	78.0+
length of neural spine	45.0+
height of neural spine	37.0+
length of body	50.5
**Thoracic vertebra F in ** **Fig. 16** **.**	
maximum preserved length	61.0+
maximum preserved height	89.0+
maximum preserved width	79.0+
length of neural spine	45.5+
height of neural spine	21.0+
length of body	45.5
**Thoracic vertebra H I and J in ** **Fig. 16** **.**	
maximum preserved length	62.0+
maximum preserved height	134.0+
maximum preserved width	118.0+
length of neural spine	42.5+
height of neural spine	67.5+
length of body	41.5
**Scapula, left**	
maximum preserved length	233.0+
maximum preserved height	169.0+
length of acromion process	97.5+
length of glenoid fossa	49.0
width of glenoid fossa	35.0
depth of glenoid fossa	8.5
**Ulna**	
Length	130.0
length without olecranon process	89.5
narrowest length of shaft	27.5
width of shaft at the narrowest point	18.5

Dimensions follow Fordyce et al. [82] and Perrin [33]. Measurements are rounded to the nearest 0.5 mm. For skull and mandible, distances are either horizontal or vertical, unless identified as point to point.

#### Material

Only the holotype is known: OM GL 421 (formerly B.J. Marples collection G.54.3 in the Department of Zoology, University of Otago, and later OM C.75.27), comprising the skull, left periotic, right bulla, mandibles, four isolated teeth, atlas, axis, three other cervical vertebrae, seven thoracic vertebrae, two epiphyseal discs, four ribs, left scapula and left radius.

#### Type locality

The specimen is from Trig Z, Gards Road, Waitaki Valley, Otago, South Island, New Zealand (Fig.1). NZMS 260 140: 146975. See details above.

#### Horizon

Probably the upper part of the Miller Member, Otekaike Limestone (Fig.1). See details under Geological Setting, above.

#### Age

The age is Waitakian stage, latest Oligocene, ≥23.9 Ma. See details under Geological Setting, above.

#### Diagnosis

*Otekaikea marplesi* is a heterodont odontocete with: a skull of medium size (bizygomatic width 257 mm, cranial length 197 mm); procumbent apical teeth; pterygoid sinus system restricted to the basicranium, without orbital fossae; a remnant intertemporal constriction with parietals exposed laterally; depressed supraoccipital; prominent condyles; robust zygomatic processes; and unfused cervical vertebrae. *Otekaikea* differs from other archaic Odontoceti including Xenorophidae, *Simocetus*, *Agorophius*, *Patriocetus*, *Prosqualodon*, Squalodontidae, *Waipatia*, and *Papahu*, in the unique combination of apomorphies involving: broad dished face; elevated nodular subrhomboidal nasals and elevated frontals; smooth-surfaced premaxillary sac fossae without prominent premaxillary sulci developed posteriorly; premaxillae strongly bifurcated posteriorly, associated with bilateral posterior accessory foramina and elevated crests on the maxillae; periotic with long slender parallel-sided posterior process, and sharp apex of anterior process. Shares with *Waipatia maerewhenua*: maxilla and occipital partly separated by parietal; flat dorsal surface of periotic; long posterior process of the periotic; and poorly developed ventromedial keel of the bulla. Shares with Squalodontidae, *Prosqualodon*, and Platanistoidea: scapula with reduced coracoid process. Differs from Squalodontidae and *Prosqualodon* in lacking large robust heterodont cheekteeth. Differs from *Notocetus*, *Squalodelphis* and Platanistidae in lacking: orbital fossae for extensions of pterygoid sinuses; thickened maxilla or elevated maxillary crest over orbit. *Otekaikea marplesi* differs from crown odontocetes other than Platanistoidea in lacking: the deep facial fossa, enlarged pterygoid sinus fossae, and enlarged posterior process of the bulla of Physeteridae, Kogiidae, and Ziphiidae; the anterior sinus fossa, an apex of the postglenoid process that is dorsally higher than the post-tympanic process, medially located aperture for the cochlear aqueduct, and strongly developed crest between the infraspinous fossa and teres fossa of Eurhinodelphinidae; and the orbital fossae for extensions of pterygoid sinuses, parabullary ridge of periotic, and saddle-shaped involucrum of the bulla of Delphinida.

#### Etymology

Dickson [18] stated that the species name honors the Professor of Zoology at the University of Otago, Brian John Marples (1907–1997).

### General description

Descriptions are based on the right or left side, whichever is more informative, with differences between them mentioned only if directional asymmetry is evident. Morphological terms follow Mead and Fordyce [28] for the skull; postcranial terms mainly follow Flower [29], and Evans and Lahunta [30]. Measurements are in Table 1–3.

#### Body size

The body size of *Otekaikea marplesi* can be inferred by the Pyenson and Sponberg [31] formula for stem Platanistoidea, Log(L) = 0.92*(log(BIZYG)-1.51)+2.49. Here, BIZYG (the bizygomatic width) of *Otekaikea marplesi* is 25.7 cm, giving a reconstructed body length of 2.5 m for the species. This is a similar size with modern *Platanista gangetica*: 2.6 m in adult female; 2.2 m in adult male, and a body mass to at least 85 kg [32]. The estimated body size of *Waipatia maerewhenua* (around 2.6 m) by Pyenson and Sponberg [31], based on Pyenson's direct study of the holotype, is just slightly larger than *Otekaikea marplesi*.

#### Cranial topography

**Figure 18:**
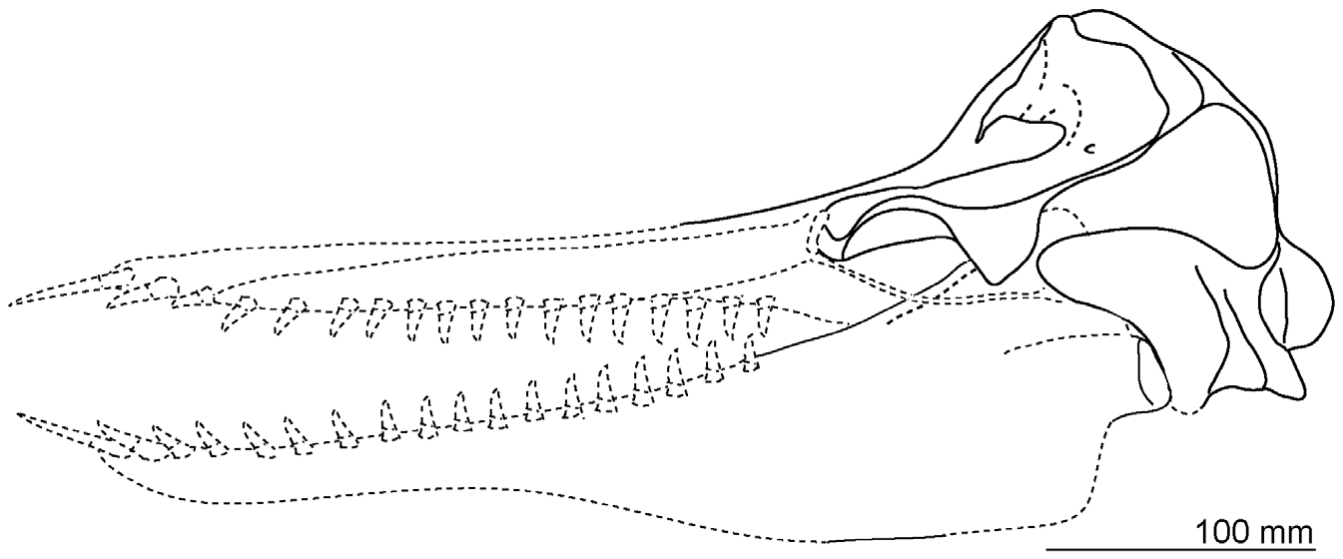
Reconstructed skull of *Otekaikea marplesi*. The anterior of the rostrum and mandibles were reconstructed based on the sister taxon, *Waipatia maerewhenua*.

The cranium (Fig. 2–8) is nearly complete. However, most of the rostrum is missing “because the skull lay with the rostrum projecting from a cliff face” [18], and as a result, condylobasal length is unknown. The jugals, pterygoids and most of the palatines are also missing. The face has a wide hexagonal shape in dorsal view, is flat anteriorly, and rises posteriorly. Asymmetry affects the nasals, frontals, premaxillae and maxillae moderately, with a left-skew (assumed to be original directional asymmetry) that is more more marked than in *Waipatia maerewhenua*. In lateral view (Fig. 6), the posterior part of the face has steep medial and posterior walls involving both maxilla and premaxilla, and these walls form a strongly elevated but anteroposteriorly short vertex comprised of the maxillae, premaxillae, nasals and frontals. The temporal fossa is diamond-shaped and mostly visible in dorsal view, in spite of the laterally expanded temporal crest. The indistinct temporal constriction lies slightly posterior to the lateral junction of the supraoccipital with the facial bones. The external nares open from a subvertical narial passage, with the internal nares opening ventrally about level with the postorbital processes. At the level of the nasals, the face is up to 45–50 mm deep. The nuchal crest is prominent and at the same level as the frontal, with adjacent depressions on the frontals, parietals and occipital. There is a wide floor for the temporal fossa on the squamosal. The temporal fossa has been squashed just slightly and the anterior tip of the squamosal is displaced upward. In the reconstruction (Fig. 18), the anterior tip of the squamosal is kept just posterior to the postorbital process, and the temporal fossa also is heightened dorsoventrally. The orbit is short and arched, with a relatively thin preorbital process, above which the frontal is almost overlapped by the maxilla.

#### Ontogenetic age

Most sutures are clear to the eye. The following features suggest that *Otekaikea marplesi* might be nearly adult, equivalent in ontogenetic age to stage V (a sexually mature but physically immature adult), using the stages proposed by Perrin [33] for *Stenella attenuata*: the nuchal crest is developed; epiphyses on each cervical vertebra are fully fused to the body; all of the preserved thoracic vertebrae have fused anterior epiphyses, five of seven thoracic vertebrae have unfused posterior epiphyses; all articular surfaces on the skull, mandibles, vertebrae and scapula are ossified and smooth; and the distal epiphysis of the ulna was not fused, and has been lost. Fused sutures and chondroses include: the frontal-orbitosphenoid; the basisphenoid-orbitosphenoid; and the vomer-basioccipital. Unfused sutures include the following: the premaxilla and maxilla on the rostrum; the maxilla-frontal; the maxilla-lacrimal; the inter-narial, the naso-frontal, the naso-premaxillary; the naso-maxillary; the interfrontal; the squamosal-parietal on the right side; the alisphenoid with the basioccipital, and the orbitosphenoid and squamosal.

#### Rostrum

Most of the anterior part of the rostrum is lost. The preserved posterior part of the rostrum, at the broken cross section, is flat and wide on the right (around 37 mm in depth and 105 mm in width) at its base, at the antorbital notch (Fig. 2 and 3). The antorbital notch is not preserved but a small part of the medial margin is preserved on the maxilla. At this point, in anterior view, the vomer is thick-walled and slightly curved laterally, and has a strongly convergent ventral part, almost a V-shape; judging from most other odontocetes, the maxilla originally covered the ventral, lateral and dorsal surfaces of the vomer. The premaxilla overlies the maxilla with a distinct unfused suture, and is thin (about 4 to 5 mm) in the broken section. Because of damage, the shape of the maxillary flange, if any, and more-lateral margin of the rostrum are not known.

#### Premaxilla

Each premaxilla overlies the maxilla anteriorly as a flat plate, with sutures clear in broken section. Posteriorly, the premaxilla thickens and rises upward, with the posterior part of the ascending process noticeably steep in lateral view. An internarial constriction is formed by the premaxilla and the maxilla, at the narrowest part of the mesorostral groove and the premaxillary sac fossa. Posteromedial to the right premaxillary sac fossa, there is a suboval foramen (not the premaxillary foramen); a comparable foramen is absent on the left. The premaxillae are asymmetrical; the left premaxillary sac fossa is a clear oval depression, while the right fossa is only weakly depressed. However, the width of right and left premaxillae at the level of the nares is the same. There is no evidence of the premaxillary foramen, posteromedial sulcus, posterolateral sulcus, or prenarial triangle; however, the foramen and one or more sulci, and commonly the triangle, are characteristic of odontocetes, and in this case must have been present in the more-anterior lost rostral part of the premaxilla. In dorsal view, the lateral edge of each premaxilla is thickened and has an anteroposteriorly elongated ridge, not, however, associated with a posterolateral sulcus. The postorbital width (127 mm) is level with the nares. Slightly posterior to the level of the nares, on the right and left sides, a single posterior accessory foramen opens posteromedially, between the maxilla and the posterolateral plate of the premaxilla; the foramen is anteroposteriorly long, elliptical, and open posterodorsally. (Posterior accessory foramen is used in the sense of Vélez-Juarbe and Pyenson [34] without implying homology between the structure as seen in Monodontidae and *Otekaikea marplesi*). Small slits on the anterolateral wall of the posterior accessory foramen are 12.9 and 5.1 mm deep on the left and right respectively. The premaxillary cleft (Fordyce, [10]) forms a notch at the posterior end of the premaxilla on the right, but further extent is uncertain because of surficial damage to the premaxilla. Posteriorly, each posteromedial splint reaches the nasal and frontal, and is skewed a bit medially, where the splint is pointed and thin.

#### Maxilla

The rostral part of this element is lost (Fig. 2 and 3). Contacts with the frontal and lacrimal are clear in most places. Both antorbital processes are broken, but the area is generally flat, and there is a very deep crescent-shaped depression on lateral margin of the maxilla, which opens laterally near the antorbital notch, perhaps marking a lateral opening of the dorsal infraorbital foramen. Anteriorly, in broken section, the maxilla and the vomer are not fused; the maxilla here overlies the dorsal edge of the vomer.

Dorsally, the maxilla is exposed slightly medial to the premaxilla and forms a small exposure (but not a full-sized maxillary intrusion, *sensu* Arnold and Heinsohn [35]) at the anterior end of the nares, in the mesorostral groove. The maxilla on the supraorbital process is flat, smooth, and not obviously thickened. There is no maxillary ridge or pneumatic maxillary crest *sensu* Mead and Fordyce [28] (cf. *Platanista gangetica* and fossil Platanistidae), and there is no evidence ventrally of a dorsally excavated preorbital sinus fossa. Slightly anterior to the antorbital notch dorsally, there is a clear ridge, which runs anteromedial to posterolateral; whether this is a rostral ridge associated with rostral muscle is uncertain. The maxilla forms the anterior edge of the supraorbital process, but does not cover the frontal anterolaterally. Laterally, the maxilla is flat across the whole supraorbital process. At least one dorsal infraorbital foramen opens on the right side around 40 mm anterior from the nuchal crest and 30 mm medial from the lateral margin of the maxilla. The large, rounded foramen (9.3 mm diameter) opens into a sulcus toward the posterolateral edge of the maxilla, supplying vessels and nerves to the facial muscles. (The region of the left foramen is damaged and infilled with plaster). Posteromedially, the squared apex of the maxilla meets the frontal at the vertex. Posteriorly, the maxilla contacts the supraoccipital, forming the base of the nuchal crest. The posterior region of the maxilla is very steep posteriorly and medially. Just lateral to the nasal and frontal, the medial border of the sigmoidal crest on maxilla (or maxillary crest) rises abruptly.

In ventral view (Fig. 3), the maxilla preserves few details. The maxilla forms the anterior and medial borders of the infraorbital foramen, with the frontal at the medial wall. There is no infraorbital plate or maxillary tuberosity; the maxilla does not contribute to the anterior wall or floor of the orbit. The broken section on rostrum shows a transversely wide canal from the infraorbital foramen, probably supplying the most anterior dorsal infraorbital foramen.

#### Palatine

Ventrally, incomplete palatine covers the maxilla, just slightly posteromedial to the infraorbital foramen, and connected with the frontal posteriorly (Fig. 3). The moderately preserved left palatine is a dorsally curved plate with a shallow groove on its anterior part, possibly a sulcus from the greater palatine foramen. The medial part of the palatine contributes to the anterolateral wall of the nares. Laterally, the palatine shows some of the palatal crest located just medial to the infraorbital foramen. The posterolateral part has a smooth shallow depression, perhaps for the pterygoid sinus fossa.

#### Nasal

Each smooth, flat, nodular nasal has a subrhomboidal outline, elongated anteromedially to posterolaterally (Fig. 2). The nasals are asymmetrical; the right has a wider anterior border. The internarial, nasofrontal, nasopremaxillary and nasomaxillary sutures are prominent, deep, and wide. Each nasal's anterior edge lies far posterior to the level of the antorbital process, almost level with the posterior end of the postorbital process. The anteromedial angles project strongly, while the posteromedial angles are displaced forward by the narial processes of the frontals. The anterior portion of each nasal is thicker than the posterior portion.

#### Ethmoid

The ethmoid is used in the sense of Mead and Fordyce [28], but note that Ichishima [36] suggested that the ethmoid might be absent in odontocetes. Morphological terms of the ethmoid follow Godfrey [37].

Hoch [38] described this specimen's ethmoid region. Further preparation now shows more detail (Fig. 4). The profile of the ethmoid is uncertain but the element is assumed to form the posterior wall of the nasal passage, dorsoventrally between the vomer and nasals. The ethmoid has a pair of large and strongly curved crescentic foramina, beside which each elliptical olfactory recess opens to the brain case. Between the foramina, there is a thin median ridge, which is assumed to be mesethmoid. On each side, lateral to the olfactory foramen, there is a large nodule, possibly a medial exposure of the frontal. Lateral to the nodule, in turn, there is a small exposure of presumed maxilla. This area is poorly preserved and identifications are provisional.

#### Vomer

The vomer is v-shaped in broken anterior section, distinctly robust and thick laterally, thin ventrally, and is open ventrally along the midline, probably because of damage (Figs 2, 4). In the broken cross section, the vomer is 45.7 mm wide dorsally and a maximum of 32.3 mm deep. In ventral view (Fig. 3), the posterior part of the vomer ends around the mid-point of the medial lip of the nares, forming the posteromedial portion of the nares; in life, the vomer presumably extended back toward the basioccipital. Ventrally, the vomer carries two tear-shaped flat areas, which might be sutures for the maxilla (this structure occurs in a young individual of *Cephalorhynchus hectori*, OU 22712). Posteroventrally, the ventral surface is damaged and rough-surfaced. In ventral view (Fig. 3), the posterior part of the vomer widens around the mid-point of the medial lip of the nares, forming the posteromedial portion of the nares and ends as covering on the basisphenoid.

#### Lacrimal

The damaged right lacrimal is exposed in dorsal view (Fig. 2) as a mediolaterally long bar-like structure, which is dorsoventrally, thin (12.0 mm) and transversely long (<17.9 mm). The remains of the lacrimal suggest that the bone covered the anterior border of the frontal as in *Papahu taitapu*, and formed the posterior wall of the antorbital notch, which now shows a fibrous surface at the presumed suture for the lacrimal. Details are uncertain for the medial end of the lacrimal, where the element is covered by the maxilla. The jugal is not preserved. There is no evidence of a lacrimal foramen or groove.

#### Frontal

Each frontal contributes a large surface to the orbit, and a small exposure at the vertex. Dorsally, the frontals are bounded by the nasals, premaxillae, maxillae and supraoccipital; there is a clear interfrontal suture, which is concave to the right, so that the frontals show slight asymmetry. Each frontal is short, trapezoidal, and nodular, widening posteriorly. The dorsal surface is generally level with the adjacent nasal, but anteriorly in each, the surface is strongly depressed in the center, and both medial and lateral edges project anteriorly. Medially, each frontal has an elongate narial process that extends slightly between the nasals. Further laterally, the frontal is covered by contact of the maxilla with the supraoccipital. The parietal margin and the external surface are extremely thin and form the dorsal part of the temporal crest.

Ventrally, the frontal forms the anterodorsal wall of the braincase and most of the deep and short orbit. The elevated and prominent preorbital ridge arises from the preorbital process laterally, and becomes elevated and narrow toward the posterior margin of the narial passage. The frontal contributes the posterior and anterior margins of the infraorbital foramen. The right frontal preserves a narrow, oval, presumed optic canal. The posteriorly-concave postorbital ridge is very sharp and high, and connects with the postorbital process, which is a blunt triangular ventral projection as seen in lateral view. Between these ridges, the roof of the orbit is short, deep, and lacking any fossae for sinuses or muscles. Each frontal has two foramina for the frontal diploic vein on the ventral triangular surface of the orbit, each opening into a slender sulcus almost in the center of the orbit. The suture with the orbitosphenoid (below) is fused.

#### Parietal

The parietal is exposed dorsolaterally as a triangle between the frontal and occipital. Below the intertemporal constriction, the parietal forms the lateral wall of braincase, which is strongly concave medially and provides a large dorsoventrally deep temporal fossa. Anteriorly within the temporal fossa, as seen in ventral view, the parietal has an irregular thin flange that extends forward to encroach the frontal. This condition occurs in *Pomatodelphis inaequalis* USNM 187414, and in undescribed specimens OU 22306 [39], [40] and OU 22540, as a rectangular flange of the parietal. It is not clear whether the flange is a neomorphic structure, or is homologous with that part of the parietal that supports the posteromedial part of the supraorbital process in archaeocetes. Here, the parietosquamosal suture is clear and sigmoidal. There is no postparietal foramen. Details of the parietal are lost anteroventrally, near the orbit and dorsally with the frontal.

Ventrally, each parietal is present in the basicranium as a small exposure medial to the squamosal and lateral to the basioccipital (Fig. 7), similar to the situation in *Waipatia maerewhenua*. The right parietal is better-exposed, probably caused by burial-related disarticulation of the squamosal, with the outer margin rotated up. The left parietal retains a clear and unfused suture medially with the sheet-like basioccipital and the alisphenoid anterolaterally. The parietal and basioccipital are sutured, forming a bridge between the foramen ovale anteriorly (still partly preserved on the left) and the cranial hiatus posteriorly. On the left, the cranial hiatus is wide (19.3 mm, left side) and large (10.8 mm long, left side). It is not clear what foramina might be present along the parieto-squamosal suture here.

#### Squamosal

In dorsal view, a wide (34.4 mm at the subtemporal crest) and short zygomatic process parallels the axis of the skull at the greatest width of the skull, posterior to the orbit. The anterior end reaches to the level of posterior end of the nasals and still far from the postorbital process (15.2 mm gap between the processes); in life the zygomatic and postorbital processes were probably closer. On the lateral surface, a long groove, the sternomastoid muscle fossa, lies between the external acoustic meatus and the post-tympanic process, extending forward to the anteroposterior midpoint of the zygomatic. The fossa is 53.9 mm long; adjacent, the supramastoid crest is quite distinct. The temporal fossa is exposed little in lateral view, is more obvious in dorsal view, and is widely open to posterior view where the temporal crest forms a near-horizontal flange at the posterior of the squamosal fossa, overhanging the exoccipital. The sternomastoid muscle fossa has a posterolaterally-oriented angle, which is formed by the supramastoid crest. Dorsally, medial to the angle of the sternomastoid muscle fossa, the posterior border of the temporal fossa sweeps inward then rises to connect with the nuchal crest of the exoccipital. On the dorsal surface of the temporal fossa, a sigmoidal suture between the squamosal and parietal is apparent.

Ventrally, the zygomatic process has a wide and shallow mandibular (glenoid) fossa, with a straight lateral portion and a strongly curved internal face. Each postglenoid process has lost the tip, but enough remains to show that each was anteroposteriorly thin, not markedly expanded laterally, and sloped obliquely posteroventrally. The tympanosquamosal recess descends down the face of the postglenoid process. The recess is short next to the glenoid fossa, becomes indistinct about 32 mm anterior to the anterior meatal crest (left side), and lacks an excavated fossa further forwards on the zygomatic process. Posteriorly, the recess is limited by the low but strong anterior meatal crest, which descends down onto the postglenoid process. The posterior part of the tympanosquamosal recess has an indistinct oval depression, perhaps the sigmoid fossa (*sensu* Geisler et al. [41]: 16), bounded anteriorly by a small anterior transverse ridge (*sensu* Fordyce [42]: 203). The apex of the spiny process is damaged and dorsoventrally thin on its base. On the left, the tip of the spiny process passes into a flange that descends ventrally into the start of the falciform process.

Anteriorly, the squamosal-alisphenoid suture is unclear (Fig. 7). The subtemporal crest is smooth and the falciform process is well developed. The distal end of the falciform process is damaged but it shows a thick base, especially thick posteriorly. The falciform process is long (22.6 mm) and slightly sigmoidal at the broken section.

The periotic fossa is developed on a robust, medially-extended flange of squamosal posterior the level of the falciform process. The periotic fossa lies posterior to the base of the falciform process, is mostly encircled by the squamosal, and is formed by the parietal at its medial border. The border on the parietal is flattened to fit the dorsal crest of the periotic.

The periotic fossa has anterior and posterior portions that are separated by a weak, indistinct supratubercular ridge, 7.3 mm anterior to the spiny process. Anteriorly, the periotic fossa descends onto the oblique posterior face of the falciform process, forming a long shallow surface to fit the anterior process of the periotic. Only part of the parietal and the posterior area of the falciform process appose the periotic; for the most part, the squamosal and the periotic are widely separated. In the middle of the anterior portion, a small fissure originates, running to the foramen ovale, which is an anteromedial-posterolaterally long elliptical foramen (5.8 mm, long diameter; 4.3 mm, short diameter). Anterior to the periotic fossa, there is a small tubercle by the foramen spinosum.

The posterior portion of the periotic fossa is wider (18.3 mm wide and 8.2 mm long) and deeper than the anterior portion. The posterior portion and the suprameatal pit is divided by an anteroposteriorly long ridge. The lateral wall of the suprameatal pit has a tiny foramen. Posteriorly, the periotic fossa is separated from the exoccipital by an anteroposteriorly thick wall. The lateral end of the wall forms the post-tympanic process.

The external acoustic meatus is very narrow and shallow, and widens a little laterally and ventrally: it has a steep anterior wall. Medially, the meatus is separated from the tympanosquamosal recess by a distinct but poorly developed anterior meatal crest. The posterior meatal crest is weaker than the anterior meatal crest. Behind the posterior meatal crest, the post-tympanic process forms the posterior of the squamosal, and provides a small suture for the posterior process of the bulla. The posterior end of the squamosal is transversely almost straight, with a widely open suture with the exoccipital. There is no evidence for a groove or notch for the facial nerve.

#### Basioccipital

This element has broken margins, and is crushed up into the braincase where it is supported by matrix (Fig. 3). Ventrally, the basioccipital is a trapezoid; it is not clear whether its anterior border was an open or, as expected, fused synchondrosis with the basisphenoid. The basioccipital crest is weak on its anterior part, and is thin and strongly projected laterally on its posterior part. There is no clear facet for the posterior of medial lamina of the pterygoid. Laterally, the basioccipital contributes to the anterior and medial margins of the posterior lacerate foramen. Medially, the posterior part of the basioccipital crest has a well developed and rounded muscular tubercle. Between the crests and tubercles is a narrow flat area without obvious muscle insertions.

#### Supraoccipital and exoccipital

Posteriorly, the supraoccipital is roughly squared, and depressed sagittally. A dorsal view shows the strongly developed and rounded dorsal part of transverse nuchal crest and the perpendicular straighter lateral border with the parietal. The dorsal condyloid fossa is wide (63.9 mm) and curves over the condyle (in posterolateral view). Its medial end is just above the condyle. Laterally, the dorsal condyloid fossa descends ventrally, to a point ventral to the posterior end of the temporal fossa.

Each occipital condyle has a smooth and small surface, is semicircular in lateral view (more sharply curved ventrally than dorsally), is less obviously curved horizontally (dorsal view), is somewhat elliptical in posterior view, and has a very short pedicle that is almost as wide as the condyle. The foramen magnum is elliptical, wider than high (35.1 mm wide and 20.8 mm height), and slightly arched dorsally; the profile may result from dorsoventral crushing. The intercondyloid notch is U-shaped.

Posteriorly, the exoccipital and the supraoccipital are fused completely, but the exoccipital shows a clear border with the squamosal, which is a result of post-mortem movement of the bones. The temporal crest is developed posteriorly, but rounded and indistinct anteriorly. Laterally, the exoccipital is thin. Its acute dorsolateral margin projects forward toward the neck muscle fossa of the squamosal, while the straight outer margin descends steeply. There are strong lateral and ventral edges, the latter thickened to form a robust paroccipital process. Both distal apices are damaged; more-medially, each has a flattened oval surface facing slightly forward. There is no evidence of a fossa for the posterior sinus, and no obvious articulation for hyoid elements. The jugular notch is missing, presumably lost when the basioccipital broke away, the hypoglossal foramen is not observed, and the contribution, if any, of exoccipital to the basioccipital crest is uncertain. Ventrally, at the exoccipital-squamosal suture, there is a large transverse groove, which connects with the posterior lacerate foramen medially.

#### Alisphenoid

The thin alisphenoid forms part of the subtemporal crest laterally. Ventrally (Fig. 7), the large alisphenoid is posterior to the frontal, anterior to the squamosal and lateral to the basisphenoid; its sutures are clear, except with the squamosal. Anteriorly, the alisphenoid has a small, long, rounded anterolateral projection toward the margin of the subtemporal crest, forming the anterior part of a large, shallow and hemispherical depression of the pterygoid sinus fossa. Just slightly posterior to the pterygoid sinus, almost in the center of the alisphenoid, there is a weak transverse ridge marking the groove for the mandibular nerve and associated vessels between anterior end of the elliptical foramen ovale (17.6 mm long diameter, 8.3 mm short diameter) and anterior end of the falciform process of the squamosal. The groove for the mandibular nerve divides the pterygoid sinus fossa into anterior and posterior (shallower, smaller) portions. Both the lateral border of the alisphenoid and the falciform process of the squamosal form the foramen spinosum as interpreted for *Waipatia* (see Fordyce [10]). Foramina here are not really clear, but there may be four, of which the most posterior is the largest (7.8 mm long). Comparable foramina are observed widely among odontocetes (e.g., *Waipatia maerewhenua, Papahu taitapu, Mesoplodon grayi, Tursiops truncatus* and *Globicephala* sp.), but homologies are uncertain [28].

#### Basisphenoid

The basisphenoid lies posterior to both the vomer centrally and alisphenoid laterally, anterior to the basioccipital without clear borders among them. A large rounded ventral carotid foramen (5.4 mm diameter) opens just medial to the foramen ovale, best seen in left ventrolateral view.

#### Teeth

*Otekaikea marplesi* is heterodont and polydont (Fig. 8), with at least one pair of procumbent anterior teeth (tusks). Three single-rooted teeth and one double-rooted tooth are preserved separate from the jaws. Tooth positions are identified with reference to other archaic odontocetes, especially *Waipatia maerewhenua*. Features of the tusked incisor (Fig. 8, A to C) include a high smooth crown, which is worn to expose dentine and the pulp cavity, which is filled by matrix. Of note, the wear pattern around the crown is asymmetric and tear-shaped; one side is worn more than the other. In one view, the worn end is slightly curved like a shovel (Fig. 8, A). The enamel has tiny vertical fissure-like ornament. The rough-surfaced root, which appears slightly worn, is long (77.0+ mm) and becomes thickest (9.8 mm widest diameter) slightly proximal to the crown. Both proximal and distal ends taper. A slim presumed proximal part of a root (26.3 mm long and 4.3 mm widest diameter) shows massive dentine in both proximal and distal broken sections (Fig. 4, C). Originally, this piece was the proximal end of the largest incisor, which was 12 cm long (*fide* Dickson [18]).

A conical tooth (Fig. 8, D to F) preserves the base of the crown and distal part of root (11.4+ mm long). The tooth orientation is uncertain, but a small enamel slit perhaps indicates the posterior or distal face (see Fig. 8, E). In *Waipatia*, seven of 10 preserved and isolated single-rooted teeth have this slit only on the posterior part of the enamel. The slit is absent in the three other teeth. The anterior part of the root is slightly flat (5.9 mm widest diameter and 5.0 mm narrowest diameter). In posterior view, the right side has a stronger curve than the left, suggesting buccal and lingual faces respectively. On the posterior edge, there is a proximodistally long elliptical-shaped naturally worn area and a polished shiny band just proximal to the elliptical area (Fig. 8, F, in lateral view). These occlusal wear facets lie slightly buccal from the posterior axis. On an occlusal broken section (Fig. 8, D), the pulp cavity opens in the center, as a very tiny (approximately 0.1 mm) and anteroposteriorly long elliptical shape.

A conical tooth (Fig. 8, G and H) is preserved as an only distal enamel piece (19.0 mm long and 4.3 mm widest diameter) forming straight outlines, very weakly curved. Its distal end is naturally worn, exposing dentin slightly.

A double rooted-tooth (Fig. 8, I to M) has a complete crown and one of two divergent roots (14.6+ mm high). Its main denticle is a tall narrow triangle (6.7 mm high from base of the crown), slightly curved lingually, with the anterior margin a sharp slightly curved keel. Its slightly worn posterior margin exposes dentin as a long band (4.8 mm long) above a worn denticle. Whereas the buccal side has weak subvertical enamel wrinkles, the lingual side has a crenelated cingulum that spans the mesiodistal base of the crown, associated with stronger ornament more-apically on the crown. The cingulum connects with a small barely-worn denticle posteriorly posteriorly. The posterior root diverges posteriorly.

#### Periotic

The odontocete periotic is a structurally complex bone that is phylogenetically and taxonomically informative. The detailed description below should aid comparison with other taxa. Here, the orientation of the periotic is based on its original position on the skull (Fig. 9). With the periotic *in situ*, the fenestra ovalis can be seen in ventral view, but the dorsal opening of the facial canal is obscured by the pars cochlearis.

The left periotic (Fig. 10 and 11) has a long anterior process, smaller posterior process and an ellipsoid body, and a dorsoventrally inflated pars cochlearis between these processes. Both the anterior and posterior processes make a relatively narrow angle with the anteroposterior axis when the periotic is placed into original position on the skull. The anterior process is polished superficially, with some areas of pitted surface on the lateral part of the posterior process, but the presumed postmortem wear is minor and does not affect overall shape or most fine detail.

The apex of the anterior process tapers forward, and is unlike *Waipatia maerewhenua*, which has strong medial and lateral expansion. There is only one prominent angle (the sharp apex) on the anterior process, without clear evidence of the anteroventral and anterodorsal angles of more-archaic Cetacea. Judging from *Waipatia maerewhenua*, the sharp apex is not homologous with the anteroventral angle. The difficulty of recognizing the anteroventral angle arises because an often-faint anterior keel generally links the anteroventral and anterodorsal angles, but the region is smoothly rounded on *Otekaikea marplesi*. The anterodorsal angle is also indistinct and rounded. In lateral view (Fig. 10 and 11, B), the anterior process is prolonged anteroventrally, and is deep and somewhat laterally compressed, without the widening seen in *Waipatia* or squalodontids. There is a deep and strongly curved parabullary sulcus [new term; see below] laterally on the anterior process, homologous with the so-called “anteroexternal sulcus” on *Waipatia maerewhenua* (see Fordyce [10]: Fig. 11c; see also Mead and Fordyce [28] and discussion below). The anteroexternal sulcus, *sensu stricto*, in Cetacea is shallow, and runs transversely from just anterior to the lateral tuberosity toward the anterior end of the dorsal crest. The medial surface of the anterior process has a weak imperforate tubercle; in *Waipatia*, the tubercle has a vertical canal. The anterior bullar facet is a shallow groove, about 7.5 mm long, with raised parallel-sided margins (4.5 mm width). The fovea epitubaria is occupied by a slightly damaged and moderate sized accessory ossicle, which is preserved between the anterior bullar facet and the mallear fossa. The ossicle has a rounded and massive projection on its medial side. The medial projection has a shallow groove on its margin, and is also widely grooved anteroposteriorly on its dorsal face, possibly forming a sulcus for the tensor tympani muscle. The lateral subvertical plate of the accessory ossicle has a flat ventral face. There are tiny foramina, one at anteromedial and two at posteromedial positions on the accessory ossicle.

The dorsal surface of the periotic has a prominent dorsal crest, bounded laterally by a long anteroposterior groove (17.6 mm). Ventral to the dorsal crest, there is an anteroposteriorly deep groove and a pit, just anterior from the internal acoustic meatus. Between the anterior process and pars cochlearis is the anterior incisure [28]; in older literature [43], this groove was termed the groove for the tensor tympani muscle. From the anterior part of the anterior incisure, a deep straight groove runs posterior to the weak tubercle on the medial surface of the anterior process. The posterior process has a deep mediolaterally long groove on its dorsal surface.

The pars cochlearis is hemispherical ventrally, mediolaterally compressed and long relative to width. The internal acoustic meatus opens on the center medially. Anterior to the internal acoustic meatus, a very tiny foramen for the hiatus Fallopii opens in the straight groove from the anterior incisure. The internal acoustic meatus is open, posteriorly wider, and tear-shaped (maximum length; 8.2 mm). The meatus contains four foramina: the proximal opening of facial canal; the foramen singulare; the spiral cribriform tract; and the area cribrosa media. There is no anterior meatal pit (see Mead and Fordyce [28]; 112) next to the proximal opening of the facial canal. In the anterior part of the internal acoustic meatus, a low thick transverse ridge separates the large and rounded proximal opening for the facial canal (2.2 mm) from the foramen singulare. The foramen singulare is rounded and smaller than in the unnamed archaic Odontoceti USNM 205491 (see Fordyce et al. [44]) but is larger than in other platanistoids such as *Waipatia maerewhenua, Notocetus vanbenedeni*, and *Phocageneus venustus*. Between the foramen singulare and the spiral cribriform tract, there is a strongly and highly developed crest. This crest bounds a large (anteroposterior length; 5.4 mm), deep and rounded area, which is shared by two foramina - the spiral cribriform tract and the small pit of the area cribrosa media. The spiral cribriform tract has a rounded opening and anti-clock wise screw structure.

The fenestra rotunda is circular and small (transverse diameter; 2.1 mm) and has a small nodule with a shallow slit, just posteromedial to the opening of the fenestra rotunda. The aperture for the cochlear aqueduct is rounded and small (1.4 mm), and opens medially in a transverse sulcus well below the adjacent lip of the meatus. The small aperture for the vestibular aqueduct is transversely long and elliptical (2.3 mm). Just ventral to the medial margin of the internal acoustic meatus, there is a weak groove that might be the median promontorial groove (see Mead and Fordyce [28]; 122). The caudal tympanic process, or posterior cochlear crest, is mediolaterally thin and projects posteriorly between the fenestra rotunda and the fossa for the stapedial muscle. A cavity on the posterior surface, between the body and cochlear portion, emphasizes the caudal tympanic process in ventral view.

On the body of the periotic, ventrally, the mallear fossa is deep, wide and slightly reniform in shape. It has distinctive anterior and posterolateral boundaries and it carries two shallow foramina at the junction of its anteromedial margin with the accessory ossicle. *Waipatia* has a foramen on the anteromedial part of the mallear fossa, but *Otekaikea marplesi* does not. The lateral tuberosity is weakly developed, and does not project beyond the border of the periotic; when the periotic is articulated, the lateral tuberosity follows the thin edge of the squamosal leading to the falciform process. The position of the lateral tuberosity lies more dorsally than the margin of the falciform process. A distinct facial crest arises at the base of the mallear fossa and descends posteriorly to the apex of the posterior process. A small indistinct depression lateral to the crest, at posterior part of the mallear fossa, may be the fossa incudis. Further posteriorly and lateral to the facial crest, the large hiatus epitympanicus is divided by an oblique ridge along the face of the posterior process, presumably marking the contact of the spiny process of the squamosal.

A narrow groove on the facial crest is possibly the tympanohyal sulcus, based on the position of the tympanohyal in other odontocetes (e.g., Mead and Fordyce [28]: 131). The fenestra ovalis is a small (1.4 mm) asymmetrical ellipse, more sharply curved anteriorly than posteriorly, and straight medially but broadly curved laterally. The dorsal opening of the facial canal is rounded, located slightly anterior to the fenestra ovalis. The facial sulcus runs straight back from the dorsal opening of the facial canal, separated by a fine crest from the more-dorsal stapedial muscle fossa and a sharp crest from the more-ventral tympanohyal groove, and becoming indistinct on the posteromedial face of the posterior process. On *Waipatia maerewhenua* and *Notocetus vanbenedeni*, the facial sulcus is weakly curved laterally. On the posterior surface of the posterior process, two grooves, an extension of the stapedial muscle fossa and the facial sulcus, run laterally together. The prolonged stapedial muscle fossa is deeper. The facial canal is dorsoventrally higher and lies ventral to the prolonged stapedial muscle fossa.

The slightly worn posterior process projects posterolaterally, and is notably parallel-sided, rectangular, long and slender (15.1 mm long and 6.4 mm wide). The posterior bullar facet is smooth but there is a parallel-sided weak ridge on its anterior part. The dorsal margin of the tip of the process is pitted, but there is no clear evidence of any fusion with the adjacent squamosal.

Dorsally, just posterior to the lateral tuberosity, is a shallow transverse groove. The groove, in turn, is bounded by a prominent ridge that rises posterolaterally to form an indistinct bulge in the profile of the periotic. This bulge may be an incipient articular rim. Posterior to the shallow transverse groove, three posteroexternal foramina are large, mediolaterally long (diameter of the largest, 0.6 mm), and elliptical.

#### Bulla

The right tympanic bulla (Fig. 12 and 13) is heart-shaped, and significantly widens posteriorly. The anterior part and base of the posterior process are damaged. For purpose of description, the dorsal view is defined as the position of the bulla when the ventral face is sitting on a flat surface (Fig. 12 and 13, A). This may differ from the life position.

Distinctive features of the bulla include the almost straight posterior medial margin of the involucrum (more apparent in mediodorsal than in dorsal view), the relatively large volume of the tympanic cavity, which is separated into anterior and posterior parts by a ridge in about mid-length, and a prominent vertical lateral furrow immediately anterior to the sigmoid process. A deep sigmoidal tympanic notch is posterior to the sigmoid process.

The condition of the anterolateral convexity, if originally present, is unknown because of damage but the area is dorsoventrally high (18.8 mm) and its outer lip anteriorly converges toward the midline without obvious inflation. The apex of the bulla is lost, and its shape is uncertain. From posterior view (Fig. 12 and 13, B), the medial profile is somewhat rectangular and its medial edge is flattened. The rectangular medial profile is formed by an angle, perhaps marking the limit for the peribullary sinus. The involucrum is thick and rugose with transverse (more-posteriorly) to oblique (anteriorly) grooves, which extend about halfway down the inner margin of the involucrum, probably marking the ventral limit of the peribullary sinus between the bulla and the basioccipital. Level with the sigmoid process, a large tubercle projects from the lateral side of the involucrum. Anteriorly, the involucrum descends steeply from a sharp crest into the tympanic cavity. On the posterior part of the involucrum, slightly posterior to the tubercle, there is a damaged base for the posterior process; the base is thick and has a smoothed V-profile, open laterally. The sigmoid process is in about the mid-length of the outer lip; the process descends ventrally to become v-shaped as it merges into the outer lip. The broken apex of the sigmoid process is crushed down onto the conical process, and the apex is lost; the process is anteroposteriorly thick (5.7 mm) and dorsoventrally thin (2.8 mm), and elliptical in section on the base. The conical process is hemispherical, slightly excavated ventrally, thick dorsoventrally (4.4 mm) and high (26.6 mm), and located ventral to the sigmoid process; its long axis is oblique, about 45 degrees from the bullar axis. The part of the tympanic sulcus on the medial face of the conical process is a horizontal indistinct groove, which posteriorly rises to meet the broken thin ridge-like outer posterior pedicle for the posterior process. Anteriorly, the tympanic sulcus cannot be traced onto the damaged sigmoid process. In ventral view (Fig. 12 and 13, C), the median furrow is mediolaterally wide, deep at its posterior origin and rapidly shallowing forwards; it is rugose, and nodular anteriorly, but lacks creases or spikes, and is anteroposteriorly long and weakly curved. Internally, three foramina appear at the broken section of the outer lip. The outer posterior prominence is mediolaterally flattened, and projects further posteriorly than the inner prominence. The posterior profile of the bulla (dorsal or ventral view) is bilobed, without a horizontal ridge between the two prominences (posterior view).

The posterior process can be articulated on both its broken pedicles, to roof the deep U-shaped elliptical foramen. When articulated thus, the posterior process is perpendicular to the conical process, directed posterolaterally at about 45 degrees. The anterior and lateral (distal) faces are damaged, so the contact or suture with the squamosal and the facial sulcus are uncertain. The posterior process is long, narrow rectangular (18.4 mm×10.2 mm), and thin. The facet for the posterior process of the periotic is smooth and weakly arched anteroposteriorly.

#### Mandibles

Both mandibles lack the anterior part, so that no alveoli remain. The left mandible is better preserved (Fig. 14, A to C). It includes the condyloid process, the mandibular fossa, and part of the coronoid process. The base of the coronoid process lies far posteriorly, about level with the angular process, and the remnants of the margin of the dorsal border rise steeply from the condyle toward the coronoid process. The short distance between the coronoid process and the condyle might suggest that the mandible in *Otekaikea marplesi* had a relatively long lever arm [45], [46].

The body of each mandible is mediolaterally flattened, dorsoventrally deep, and is slightly smoothly inflated laterally. Laterally, a ridge develops toward the condyle. There is no masseteric line on the ventral border of the angular process (right side). The condyloid process faces posteriorly and is semicircular shape and narrow in posterior view. The medial surface of the condyloid process is excavated and rugose. On the medial surface, the dorsal part of the condyloid process passes into a ridge that runs posteriorly. The mandibular fossa is large, wide and dorsoventrally high; anteroposterior length, from anterior margin of the mandibular fossa to posterior end of the condyloid process on left side, is 151 mm, and dorsoventral depth is 94 mm. The anterior margin of the mandibular fossa was reconstructed using the impression in matrix of missing bone, to give a rounded profile. On the left, the dorsal margin of the mandibular fossa is a strong ridge. The medial surface of the dorsal border is slightly concave laterally. Most of each coronoid process is preserved, although both apices are missing; the right process has most of the anterior margin, and a little of the posterior margin. The right coronoid process has a subcoronoid ridge on the lateral face, below the rising crest of the coronoid process. There is a shallow and clear groove on the coronoid crest, which is probably the dorsal border of the masseter muscle origin.

#### Atlas

The atlas (Fig. 15 A, F and K) is anteroposteriorly thick and not fused to the axis. The epiphyses are fused with the body. The slightly damaged neural spine is long and massive, and swells dorsolaterally. From dorsal view, the neural arch is concave anteriorly. The foramen for the first spinal nerve is large and rounded, and lies in the center of the neural arch. In anterior or posterior view, the neural canal is a dorsally flattened sub-circular shape. Two transverse processes are linked by a very weak dorsoventral ridge. The upper transverse process is small and projects strongly dorsolaterally, above and slightly anterodorsal to the articular surface for the axis. The lower projection is shorter but more robust than the upper transverse process, is wide (10.5 mm long) and low (9.9 mm thick) and projects posterolaterally. The condyloid facets (articular surfaces) are shallow and distinctly separated ventrally. In lateral view, the condyloid facets are oriented at around 50 degrees from the plane of the facet for the axis, suggesting that the head might be oriented downwards relative to the body axis. The bilateral articular facets for the axis are flat laterally, but the fovea for the odontoid process of the axis is deeply depressed. On each articular facet, an oblique ridge descends forward to delimit the odontoid region; the transverse ligament (see Struthers [47]) probably arose from the ridge. From the posteroventral margin of the inferior arch, a long and robust hypapophysis (ventral tubercle), broken apically, projects posteroventrally from an elongate base. The hypapophysis extends at least 10.5 mm below and 14.3 mm behind the body. In cross section, the hypapophysis is wide, dorsoventrally thin and elliptical (measured on a point 6 mm away from the body: width 15.6 mm and depth 11.1 mm).

#### Axis

The axis (Fig. 15, B, G and L) is anteroposteriorly thinner than the atlas and not fused with the adjacent vertebrae. The epiphyses are fused with the body. The slightly damaged, robust, and straight neural spine projects posterodorsally, and is anteroposteriorly long, high and mediolaterally thin. The base of the neural spine, on the posterior surface of connection with the neural arch, has a triangular depression. The mediolaterally thin neural arch is weakly rounded to laterally. A small, anteroposteriorly long, elliptical posterior zygapophysis (9.3 mm wide and 12.8 mm long) faces ventrolaterally, lateral to the neural spine. In anteroposterior view, the neural canal is ventrally wide and subtriangular in shape, and its anterior opening is slightly smaller than the posterior. Each transverse process is thick and strongly projected laterally, with a horizontal keel from the apex to the margin of the posterior epiphysis. A rounded foramen (perhaps vertebrarterial) opens just posteriorly only on the right transverse process (3.2 mm diameter); the equivalent on the left is a shallow depression.

The odontoid process and the wide and flat articulations for the atlas anteriorly share a smooth surface. The posterior surface of the body is slightly depressed and wide elliptical with small projection on the ventral margin, which forms a weak crest on the ventral surface of the body to the odontoid process.

#### Other cervical vertebrae

Three cervical vertebrae, probably the fourth, sixth and seventh (based on the postuated fit with other vertebrae), are preserved. All the vertebral epiphyses are ankylosed. These cervical vertebrae have a tiny neural spine (broken in the seventh), strongly projected transverse processes, an anteroposteriorly flattened body, which is weakly convex in the anterior transverse plane, a sagittal ventral keel on the body and a low dorsal sagittal crest separating bilateral dorsal depressions on the body. The bodies become thicker posteriorly. There is no clear evidence of a notochordal pit; the epiphyseal margins are slightly swollen presumably for the annular ligament.

The presumed fourth cervical vertebra (Fig. 15, C, H and M) has the smallest transverse process, and a long and thick neural arch, which is wider than high. Thus the neural canal is wide (39.3 mm), low (18.5 mm) and pentagonal. There is a low neural spine (11.2 mm) and posterior zygapophyses on the neural arch, which face ventrolaterally. Slightly ventral to the posterior zygapophysis, a tiny and mediolaterally thin anterior zygapophysis projects, facing dorsomedially (size of the articular face is 17.9 mm long and 10.6 mm wide). The neural arch connects with the pentagonal, thick (25.0 mm) and robust body (46.4 mm wide and 42.0 mm high at anterior). The transverse process has a laterally long elliptical large vertebrarterial foramen (12.2 mm mediolaterally and 7.4 mm dorsoventrally) with a robust ventral border and thin dorsal border.

The presumed sixth cervical vertebra (Fig. 15, D, I and N) comprises just the body and transverse processes. The body is rounded (24.6 mm long, 41.6 mm high and 50.9 mm wide anteriorly). A long transverse process (+48.4 mm) is robust at its base and thinner at its end, and slightly curved ventrally. Only the left side preserves the remnants of a distinct vertebrarterial foramen, while the right transverse process is imperforate, thin and posteriorly excavated. The ventral margin of the transverse process is robust, and keeled horizontally and ventrally, with a prominent excavation between the keels.

The presumed seventh cervical vertebra (Fig. 15, E, J and H) has an unusual large vertebrarterial foramen and long robust ventral transverse process (right side). The transverse process lengthens ventrolaterally (60.0 mm long) and its end becomes thicker and flat. From ventral and dorsal view, the transverse process is parallel sided and flattened ventrally. Its length is around 21 mm. The diamond shaped transverse foramen is wide (36.8 mm) and low (17.0 mm at the highest point). Its dorsal and ventral parts are thin, but the lateral end (the dorsal lamina or parapophysis in Rommel [48]) is thicker. An area between the lateral end (the dorsal lamina) and the transverse process (the ventral lamina in Rommel [48]) is excavated. The proportion of the neural arch is similar with the fourth cervical vertebra; the pedicles and arch are more robust, but the anterior and posterior zygapophyses are slightly distorted and exact orientations uncertain other than oblique and similar to the sixth cervical.

#### Thoracic vertebrae

Seven thoracic vertebrae and two isolated epiphyses are preserved (Fig. 16, F). All the anterior epiphyses are ankylosed but five of the seven posterior epiphyses are not fused. Each thoracic vertebra has the following features: a high, long and flat neural spine, which has a groove on its posterior surface; a wide and low neural canal; a high positioned transverse process; synovial articulations (costal foveae) for the rib on the transverse process and the body are small and large respectively; a weakly depressed anterior surface of the body; an anteroposteriorly long body; and no distinctive ridge on ventral surface of the body. On the dorsal surface of the body, there are anteroposteriorly long elliptical shaped dorsal foramina.

Thoracic vertebra A on Figure 16 is assumed to be the first thoracic because of the perfect fit with the seventh cervical vertebra. It has the widest and lowest neural canal, shortest body and anteroposteriorly shortest neural spine among the series of the preserved thoracic vertebrae. Vertebrae B and C (of Fig. 16) fit in sequence with A, and so are the second and third thoracics. The other thoracic vertebrae are of less certain sequence. Vertebra H (I and J from different views) may be the fifth or slightly posterior to that. Vertebrae D and F seem more posterior thoracic vertebrae, given the anteroposteriorly long neural spine, high pentagonal shaped neural canal and long body. Vertebra G might be most posterior preserved thoracic vertebra, with the anteroposteriorly longest base of the neural spine and the highest neural canal.

#### Ribs

One more or less complete right anterior double-headed rib (Fig. 17, I–L) is 170 mm long (chord), anteroposteriorly flat (around 10 mm in the mid shaft) and wide (around 22 mm at the same point). The heads of the rib are flat; there is a triangular and shallow cavity on the posterior surface, which runs to the medial surface of the tubercle of rib. On the shaft, the anterior and posterior surfaces are weakly swollen and flat respectively. The flattened point is the widest on the rib and the rib portion converges distally. The distal end swells mediolaterally and provides a suboval flat and rugose costochondral junction for rib cartilage, possibly for a sternal rib. A single left double-headed rib preserves only the proximal part (Fig. 17, J). It has a wider shaft and stronger angle. Thus, originally, it located more posterior than the right rib. The triangular cavity on the posterior surface is deeper, larger, and opens dorsally. The angle of rib has a stronger ridge. Its broken section shows thick periosteal bone and a cancellous core.

#### Scapula

The left scapula (Fig. 17, A–D) is generally well preserved, except for the suprascapular (dorsal) border, the anterior end of the acromion and the coracoid. There is no evidence for a cartilaginous dorsal margin. The fan-shaped scapula is thin, long and wide, with an estimated angle of the lines from the anterior and posterior borders of ≦93 degrees. In life, the scapula was probably oriented with the glenoid cavity ventrally and the suprascapular border dorsally, as assumed in this description.

The prescapular (supraspinous) fossa is very narrow but distinct. The posterior border of the prescapular fossa consists of a developed ridge with an adjacent groove for the prescapular (infraspinous) fossa. The large postscapular fossa has a smooth surface; there is a diffuse convex posterior part. The posterior convexity for the border between the infraspinatus and the teres major lies almost at the center of the posterior half of the scapula. Between the spine and the posterior convexity, there is a shallow and wide depression for the infraspinatus.

The acromion process is long, thin and parallel-sided, and projects slightly ventrally when the glenoid cavity of the scapula is sitting on a flat plane; distally, some of the margins are damaged. The acromion is 10.2 mm thick at its suboval base and around 4.0 mm on its end. The coracoid is broken, but is inferred to have been rod-like because of its small rounded broken base (8.8 mm high, 6.5 mm wide) (Fig. 17, C), as seen in the *Otekaikea*-like specimen OU 22306 which has a complete delicate coracoid. The neck of the scapula is strongly constricted. The glenoid cavity is deep and long, with an anteroposteriorly long elliptical shape.

#### Ulna

The presumed left ulna is robust proximally and mediolaterally thin distally (Fig. 17, E–H) and there are no strong grooves on its surface. The olecranon projects posteriorly and is slightly curved probably laterally (based on an examination of an undescribed ulna with humerus of *Otekaikea*-like specimen OU 22306) from proximal and distal view. The olecranon is rugose, large, and axe-shaped, and damaged distally. The proximal end of the olecranon has a crest that meets with a proximal joint for the humerus. The proximal articular face is posteriorly narrow and heart-shaped, with two projections or coronoid processes on its anterior margin. Between the coronoid processes, there is a triangular, shallow and short radial notch. The trochlear notch is strongly curved (angle 118°). Slightly distal to the coronoid processes and the radial notch, there is a transverse groove that might be the suture for both the shaft and the proximal epiphysis. The interosseous border is straight with a mediolaterally thin crest, which has a small tubercle on the distal part of the crest. On the other hand, the outer border is strongly curved from the olecranon to the distal end of the shaft. The distal epiphysis is absent. The distal end is long and elliptical (length 38.4 mm and width 21.8 mm).

### Phylogenetic analysis

The phylogenetic position of *Otekaikea marplesi* was analysed using a modified version of the matrix of Murakami et al. [11], which was designed to focus on Odontoceti phylogeny (Appendix S1, cladistic matrix in nex format; Appendix S2, cladistic matrix in TNT format). The Murakami matrix included 73 extant and extinct taxa, all named and described, and 282 characters cited and/or modified from previous studies [5], [7], [10], [35], [49]–[58].

The Murakami matrix was modified in minor ways (Appendix S3, character list; Appendix S4, list of coding modifications). Six characters were deleted from the matrix because of overlap with other characters, and/or difficulty of coding. Two modified characters were added from Fordyce [10]. In total, 278 characters were used. Three taxa were added: *Otekaikea marplesi, Papahu taitapu*, and *Squalodelphis fabianii* (the first two coded from direct examination of type specimens, the third from a cast). An archaeocete, *Georgiacetus vogtlensis*, was used as the outgroup taxon. Percentages of missing data are: for *Otekaikea marplesi* 52% (includes soft tissue characters) and 46% (excludes soft tissue); for *Papahu taitapu* 55% (includes soft tissue characters) and 49% (excludes soft tissue); for *Squalodelphis fabianii* 66% (includes soft tissue characters) and 62% (excludes soft tissue). In total, 76 taxa or OTUs (Operational Taxonomic Units) were included in the matrix. Several codings were changed from the Murakami codings, based on direct observation of *Waipatia maerewhenua, Notocetus vanbenedeni, Pomatodelphis inaequalis, Zarhachis flagellator* and *Platanista gangetica* (see Appendix S4).

Character data and tree data were managed using Mesquite 2.75 [59]. Two cladistic analyses were performed with TNT version 1.1 [60]. All characters were treated as unweighted and unordered (analysis 1), or weighted using the implied weight value K = 3 without ordering (analysis 2). Both analyses used heuristic searches of 10,000 replicates. The swapping algorithm used was tree bisection reconnection (TBR); with 10 trees saved per replication. To measure node stability, we used the decay index [61] for the strict consensus tree from the equally weighted analysis 1, and branch length for the tree from the implied weights analysis 2. After analyses 1 and 2 were completed, species in the more-diverse crown families in the Murakami et al. [11] matrix were merged for ease of illustration.

### Phylogenetic relationships

#### Analysis 1: unweighted and unordered

**Figure 19:**
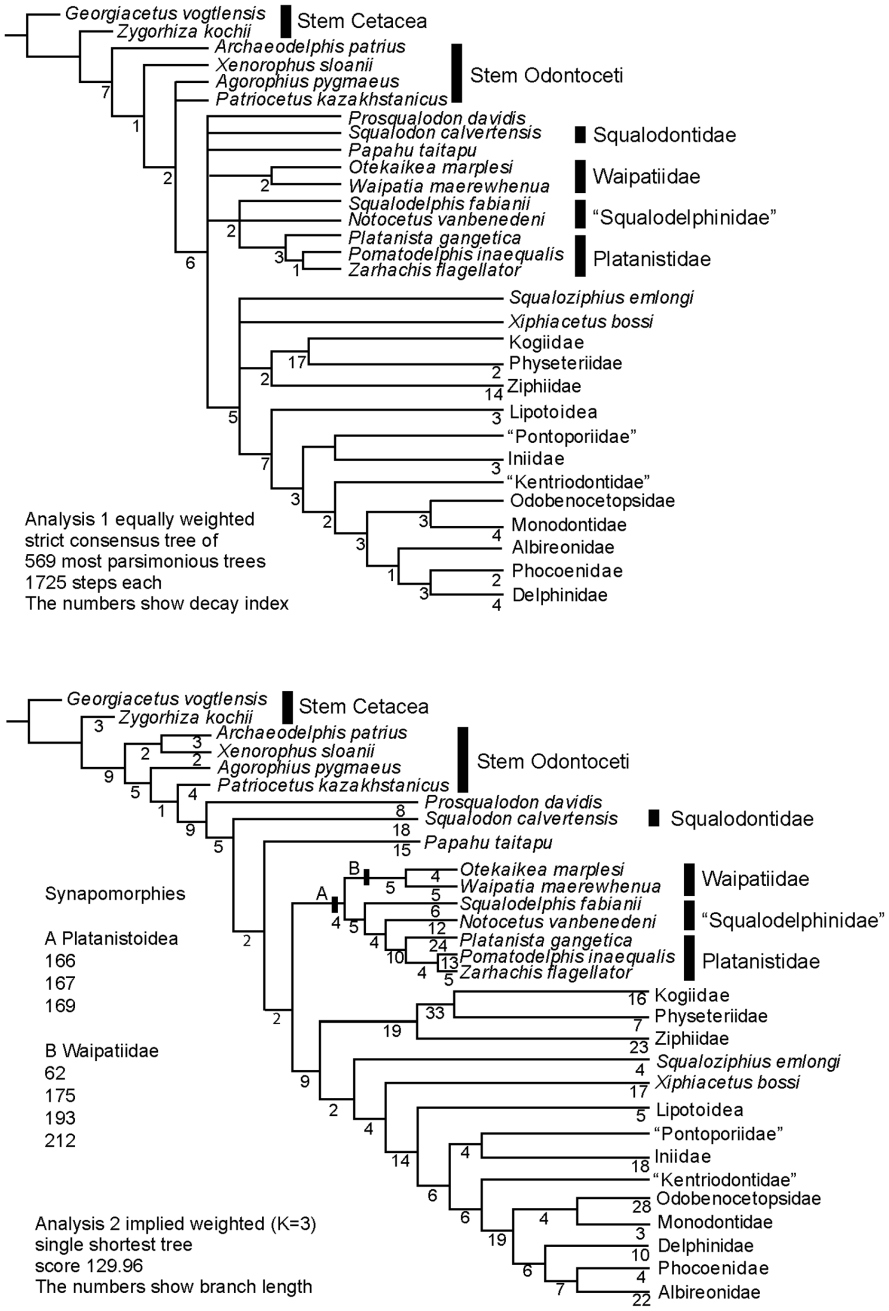
Phylogenetic analysis of *Otekaikea marplesi* and the Odontoceti. Top: strict consensus tree from equally weighted analysis 1, showing decay index. Bottom: single shortest tree from implied weighted analysis 2, showing branch length.

The first phylogenetic analysis with unweighted and unordered characters shows 312 shortest trees of 1726 steps each. The strict consensus tree (Fig. 19) shows an almost identical topology at the family level to that of Murakami et al. [11], except for the Platanistoidea (see later discussion), and the phylogenetic position of *Squaloziphius emlongi* and *Xiphiacetus bossi*. Details are discussed below.

#### Analysis 2: implied weighting

The second phylogenetic analysis with implied weighting characters recovered a single tree with a Tree Bisection Reconnection score of 129.92 (Fig. 19). The tree shows a slightly different topology for the Albireonidae from the tree of Murakami et al. [11]. Some unsolved topologies in analysis 1, above, are solved for the Platanistoidea, *Agorophius pygmaeus*, *Squaloziphius emlongi*, and *Xiphiacetus bossi*. Below, we discuss the implications of the resulting tree for: the phylogenetic position of *Otekaikea*; the Family Waipatiidae; the Family Squalodelphinidae, *Squalodelphis*, and *Notocetus*; the Superfamily Platanistoidea; and the phylogenetic position of the odontocetes *Papahu*, *Squalodon*, and *Prosqualodon*.

### Phylogenetic position of *Otekaikea marplesi*

The strict consensus tree of equally weighted analysis and the tree from implied weighting both show *Otekaikea marplesi* as a sister taxon of *Waipatia maerewhenua*. Both species are here placed in the Waipatiidae, as discussed below. *Otekaikea marplesi* is clearly morphologically different from *Waipatia maerewhenua*, and is likewise also different from the genera *Prosqualodon* and *Notocetus* in which *Otekaikea marplesi* was placed previously. Compared with *W. maerewhenua*, *Otekaikea marplesi* has a more marked asymmetry at the vertex, readily seen in dorsal view, involving: the size of the nasal and frontal; shape of the posteromedial and posterolateral splint of the premaxilla; and size of the nares. The differences are not apparent in some of the cladistic characters involving asymmetry, which are coded the same in *Otekaikea* and *Waipatia*: size of the premaxillary foramen (character 53); width of the premaxilla at the level of mid-point of the nares (character 75); size of the nares (character 79); and the cranial vertex proportion (character 98). *Waipatia maerewhenua* is more derived than *Otekaikea marplesi* in: two posterior dorsal infraorbital foramina opening on each maxilla (character 59); transversely wide nasals compared with the maximum length of the nasals (character 91); transversely straight nasal-frontal suture (character 94); squared and ventrally projected postglenoid (retroarticular) process of the squamosal in lateral view (character 115); and a small contact between the squamosal and the anterior process of the periotic (character 171). These differences between *Waipatia maerewhenua* and *Otekaikea marplesi* are considered sufficient to separate the species in two genera.

### Family Waipatiidae

The Waipatiidae hitherto contained only one species, *Waipatia maerewhenua* Fordyce, 1994. Both of the cladistic analyses here place *Waipatia maerewhenua* and *Otekaikea marplesi* as sister taxa, now included in the Waipatiidae as separate genera. The four supporting synapomorphies are: maxilla and occipital are separated by the parietal (character 62); flat dorsal surface of the periotic (character 175); long posterior process of the periotic (character 193); and poorly developed ventromedial keel of the bulla (character 212). Characters 62 and 193 involve reversals. More closely-related taxa could help to confirm character polarities and to firm up relationships within the group. In addition, *Waipatia maerewhenua* and *Otekaikea marplesi* are known from only one specimen each, with no indication of morphological variation. Of the other species mentioned as possible waipatiids, *Sachalinocetus cholmicus* Dubrovo in Siryk and Dubrovo 1970 (early Miocene, Sakhalin) [62], *Sulakocetus dagestanicus* Mchedlidze, 1976 (latest Oligocene, Caucasus) [63], and "Waipatiidae indeterminate" and "Waipatiidae?" noted by Bianucci et al. [21] (late early Miocene, Malta), might belong in the family. However, relationships have yet to be assessed by phylogenetic analysis based on direct coding of the specimens rather than from published descriptions and illustrations. *Papahu taitapu*, Aguirre-Fernandez and Fordyce, 2014 (early Miocene, New Zealand) [8] was originally placed cladistically close to *Waipatia*, but not formally in the Waipatiidae. The current phylogenetic position is discussed below.

### Family Squalodelphinidae, *Squalodelphis*, and *Notocetus*

Two nominal species of Squalodelphinidae are in the phylogenies (Fig. 19): *Squalodelphis fabianii* Dal Piaz 1917 (uncertainly later Aquitanian or Burdigalian, early Miocene, Italy) [64], and *Notocetus vanbenedeni* Moreno 1892 (Burdigalian, early Miocene, Argentina) [19]. The implied weights phylogeny shows *Squalodelphis fabianii* and *Notocetus vanbenedeni* as adjacent stem taxa of the Platanistoidea, rather than in a monophyletic Squalodelphinidae. Further, there is no support for *Otekaikea* as a genus of Squalodelphinidae. Previously, Muizon [14] proposed several apomorphies for the Squalodelphinidae, but most of these features (including those of the periotic, malleus and atlas) cannot be examined in *Squalodelphis fabianii* because they are absent or not prepared. *Squalodelphis fabianii* has more missing data than for most species considered here, in spite of it being the type species for the type genus of the Squalodelphinidae. *Notocetus vanbenedeni*, however, is better known. Comparison between *Notocetus vanbenedeni* and *Squalodelphis fabianii* shows several distinctive differences. For example, the orbit is small and shallow in *Squalodelphis*, but large and strongly excavated in *Notocetus*. The antorbital process is mediolaterally compressed in *Squalodelphis* (derived condition in character 62) but nodule-like in *Notocetus*, and the anterior end of the antorbital process is rounded in *Squalodelphis* but tapered in *Notocetus*. Finally, the postorbital process is small in *Squalodelphis* and robust and strongly projected ventrally in *Notocetus*. Whether *Squalodelphis fabianii* is best viewed as the only species in a monotypic Squalodelphinidae, or with *Notocetus vanbenedeni* as members of stem-Platanistidae, is debatable. Eventually, new material will be found to resolve the identity of the species, allowing the content of the Squalodelphinidae to be reassessed. Meanwhile, we recognise Platanistidae as the clade *Platanista* + (*Zarhachis* + *Pomatodelphis*).

Fordyce [10] used a new combination, *Notocetus marplesi*, to remove *"Prosqualodon" marplesi* from *Prosqualodon* and the Squalodontidae, and place it provisionally in the Squalodelphinidae. Cladistic analysis shows that *Notocetus vanbenedeni* differs from *Otekaikea marplesi* at genus level in characters including: elongated posterior projections of the premaxillae posterior to the nasals (character 78); wide angle of the basioccipital crest in ventral view (character 157); much larger aperture for cochlear aqueduct than the aperture for vestibular aqueduct (character 180); and slender bulla (character 198). Additional features seen in *N. vanbenedeni* but not in *O. marplesi* include: thickened maxilla above the orbit; squared anterior end of the zygomatic process; and inflated occipital shield.

Other records of nominal squalodelphinids involve taxa [14], [65], [66] for which the relationships are not clear: *Squalodelphis pusillus* Ginsburg and Janvier 1971 (Langhian, early Miocene, France) [67], *Notocetus* sp., Langhian? middle Miocene, Maryland), *Phocageneus venustus* Leidy 1869 (Langhian, middle Miocene, Virginia, USA) [68], and *Medocinia tetragorhina* Delfortrie, 1875 (Burdigalian, early Miocene, France) [69]. Of these species, *Squalodelphis pusillus* is based on an isolated tooth of uncertain relationships, and is perhaps a *nomen dubium*. The other taxa, which are early to middle Miocene and from the Atlantic rim, have yet to be analysed phylogenetically.

### Superfamily Platanistoidea

Muizon (e.g., [14], [70]) used two scapular features to define the Platanistoidea: absence of the coracoid process; and loss of the supraspinous fossa. Generally, the scapula is not well preserved in fossils, but enough specimens are known to show that scapular patterns are more complex than suggested by Muizon. Of note, the scapula in *Otekaikea marplesi* has a subcircular broken base for the coracoid process, suggesting a rod-like process as in the undescribed *Otekaikea*-like OU 22306. In other odontocetes, such as the Delphinidae, the coracoid process is a more-robust structure that provides an origin for the coracobrachialis muscle, which runs into the lesser tubercle (medial tuberosity) of the humerus. The distinctive coracoid process in platanistoids presumably reflects differences from other odontocetes in terms of humerus action, swimming habits, and ecology [71], [72].

The strict consensus tree in this study does not recover a monophyletic Platanistoidea *sensu* Muizon [14] or *sensu* Fordyce [10]. Rather, the tree shows an unresolved polytomy of *Prosqualodon* + *Squalodon + Papahu* + Waipatiidae + (Platanistidae + *Squalodelphis* +*Notocetus*) + later-diverging Odontoceti (including the Delphinida, Ziphiidae, and Physeteroidea).

The single shortest tree from the implied weighting analysis, conversely, shows a clade Platanistoidea, involving the Waipatiidae + (*Squalodelphis fabianii* + *Notocetus vanbenedeni* + Platanistidae). Platanistoidea in this sense is a stem based group, which is the lineage containing the living *Platanista gangetica* and all odontocetes more closely related to *P. gangetica* than to any other crown odontocete clade. Platanistoidea is also the most basal lineage of the crown Odontoceti. *Prosqualodon*, *Squalodon*, and *Papahu* are excluded. The clade is supported by three synapomorphies: presence of lateral groove and overall profile of periotic becoming slightly to markedly sigmoidal in dorsal view (character 166); anteroposterior ridge developed on dorsal side of the periotic (character 167); and presence of parabullary sulcus (character 169; see more discussion in morphology section). All the synapomorphies are periotic characters.

Future work on platanistoids and close taxa would benefit by access to well-preserved material of *Notocetus*, *Zarharchis* and *Pomatodelphis* – fossils which might bridge the huge morphological gap between the highly autapomorphic (disparate) living *Platanista gangetica* and more-basal stem platanistoids. Future work should also include other *Otekaikea*-like odontocetes and especially more squalodontids, for these are poorly represented in published phylogenies.

Phylogenetic position of *Papahu*, *Squalodon*, and *Prosqualodon Papahu taitapu* is known from an incomplete skull from New Zealand (early Miocene, Otaian stage, middle Aquitanian to middle Burdigalian). The cladistic analysis by Aguirre-Fernandez and Fordyce [8] placed *P. taitapu* next to *Waipatia maerewhenua* but not in the same clade. The implied weights tree of this study recognizes *Papahu taitapu* as the sister taxon to the crown Odontoceti, with *Prosqualodon davidis* and *Squalodon calvertensis* immediately more-basal. The strict consensus tree from the equally weighted analysis shows *P. taitapu*, *S. calvertensis* and *P. davidis* in an unresolved polytomy, signalling a need for more squalodontids to be included in cladistic studies.

The phylogenetic positions of *Prosqualodon davidis, P. australis* and the Squalodontidae have long been problematic. Cozzuol [73] placed *Prosqualodon* in its own family Prosqualodontidae, and mentioned that *Prosqualodon* might be related to the Delphinida. Geisler and Sanders [5] and Geisler et al. [7] recognized *Prosqualodon davidis* as a member of the stem Odontoceti. Murakami [11], [74] showed *Prosqualodon davidis* as a sister taxon of *Waipatia maerewhenua* among the Platanistoidea. Earlier non-cladistic studies placed *Prosqualodon davidis* in the Squalodontidae (e.g., [75]–[77]). A comparable phylogenetic position of *Prosqualodon davidis* was proposed by Muizon [78] who moved *Prosqualodon davidis* from the Squalodontidae to a position as the sister group of all other platanistoids, because of lack of squalodontid skull synapomorphies earlier cited by Muizon [70]. Uncertainties about *Prosqualodon* probably reflect the lack of published codings sourced from well-preserved original specimens. As for the content and identity of the Squalodontidae, our working hypothesis follows Fordyce [10] and Muizon [78]: the family is a multi-species clade that includes some nominal species of *Squalodon* from the margins of the North Atlantic, *Phoberodon arctirostris* from the South Atlantic, and probably unnamed Oligocene species from the North Atlantic (Charleston, South Carolina) and Southwest Pacific (New Zealand).

### Morphology of the periotic and squamosal

Some issues of periotic morphology require comment. There are two lateral sulci on the anterior process of the periotic in some odontocetes, one sulcus newly named in this paper. Firstly, Fordyce ([79]: 31) named the anteroexternal sulcus, based on the periotic of an archaic Odontoceti USNM 205491, in which an oblique sulcus runs from near the lateral tuberosity to the anterodorsal angle of the periotic. Secondly, the parabullary sulcus of the periotic [new term] is here defined as the sulcus that lies on the lateral surface of the anterior process of the periotic, just lateral to and roughly parallel with the anterior bullar facet. The parabullary sulcus is characteristically curved, concave dorsally. It may merge posteriorly with the anteroexternal sulcus near the lateral tuberosity. In some previous studies, the parabullary sulcus was identified and named as part of the anteroexternal sulcus (e.g., in *Waipatia maerewhenua* [10], and in the eurhinodelphinid periotic reported by Fordyce [79]: Fig. 2c).

The parabullary sulcus is normally present among the putative platanistoids of this study, except *Platanista* gangetica (see Fig. 11 A for *Otekaikea marplesi*). *Platanista gangetica* has a highly modified anterior process, sometimes with multiple associated supplementary ossicles, and it is hard to recognize sulci on the lateral face of the process. Among fossil platanistoids, the expression of the curve is variable (e.g., strong in *Waipatia maerewhenua*; weak in *Notocetus vanbenedeni* and the extinct Platanistidae). Among these taxa, the parabullary sulcus lies anterior to the anteroexternal sulcus if the latter is developed.

The lateral tuberosity and/or mallear fossa can be good landmarks to identify the anteroexternal sulcus. The anteroexternal sulcus runs from just anterior to the lateral tuberosity and/or mallear fossa, obliquely to the anterodorsal angle at the anterior end of the dorsal crest on the dorsal surface in various basal Odontoceti, basal Mysticeti, and Basilosauridae. For example, the stem odontocete *Simocetus rayi* (of Fordyce [42]; USNM 256517) has a periotic in place, which shows a prominent ventral foramen for the anteroexternal sulcus anterior to the lateral tuberosity and the mallear fossa. The anteroexternal sulcus is a prominent oblique groove in the archaeocete *Zygorhiza kochii* (Reichenbach in Carus [80]; e.g., ALMNH 2000.1.2.1, and Kellogg [43] - USNM 10855); for archaeocetes this has been called the vascular groove (Luo and Gingerich [81]). Most other Odontoceti (Delphinoidea, Ziphiidae, Physeteridae) lack a clearly developed anteroexternal sulcus, probably because of the well-developed parabullary ridge on, and/or widening of, the lateral part of the periotic.

In other taxa including *Papahu taitapu*, *Eurhinodelphis cocheteuxi* (see Lambert, 2005; IRSNB M.1856), a putative eurhinodelphinid (Fordyce [79] AMNH 102194) and *Phocageneus venustus* (USNM 21039), there is a strongly curved V- or U-shaped sulcus, open anteriorly, which might be formed from the merged parabullary sulcus and the anteroexternal sulcus. On these taxa, the sulcus has a strong angle slightly dorsal to the lateral tuberosity. The anteroexternal sulcus portion elongates toward the anterior end of the dorsal crest, as with the sulcus in some members of Platanistoidea (Waipatiidae, *Notocetus*, and Platanistidae except *Platanista*). More work is needed to establish the phylogenetic significance of the sulci among the Odontoceti.

*Otekaikea marplesi* has a suprameatal pit on the squamosal [5]. The pit receives the articular process (or articular rim) of the periotic in *Platanista gangetica* according to Muizon [14]. However, in *Otekaikea marplesi* the suprameatal pit lies slightly ventral to the small articular process, and does not receive the process. The pit is thus a large cavity, probably for soft tissue; whether vascular or part of the pterygoid sinus complex is uncertain. A similar condition is seen in *Waipatia maerewhenua*. The suprameatal pit lies just lateral to the hiatus epitympanicus of the periotic.

### Geological and geographic distribution

*Otekaikea marplesi* and *Waipatia maerewhenua* are from, respectively, the Miller Member and immediately-underlying Maerewhenua Member of the Otekaike Limestone. Graham et al. [24] showed strontium isotopic ages for these members, which - considering also the presence of *Globoturborotalita woodi* - we interpret to give *O. marplesi* an age of ≥23.9 Ma. The lowest strontium date (of Graham et al. [24]) for the Maerewhenua Member was 25.37 Ma, although the member extends below the level dated by Graham et al. The Duntroonian-Waitakian stage boundary is 25.2 Ma [24], and *W. maerewhenua* is from the upper Duntroonian [10]. Thus, *W. maerewhenua* may be 1.3 Ma older than *Otekaikea marplesi*.

*Waipatia* and *Otekaikea* represent the Waipatiidae, a basal group of platanistoids that lived in the southwest Pacific during the late Oligocene. As with any phylogenetic study on variably incomplete fossils, more could be done to firm up relationships, age ranges, and geographic distribution with more crownward and geologically younger marine platanistoids in the same clade, especially *Squalodelphis*, from the Atlantic-Tethys.

## Conclusions

The archaic marine dolphin *"Prosqualodon" marplesi* Dickson 1964 (Otekaike Limestone, latest Oligocene, ≥23.9 Ma) from the Waitaki region of New Zealand is re-described and placed in the new genus *Otekaikea*. The single known specimen adds to odontocete morphological and taxonomic diversity during the late Oligocene in the Southwest Pacific Ocean. *Otekaikea marplesi* is readily distinguished from the stratigraphically older and related *Waipatia maerewhenua*; both are here placed in the Waipatiidae and in turn in the Platanistoidea. *Otekaikea marplesi* and *W. maerewhenua* are comparable in body size. *Otekaikea* belongs in the Platanistoidea, along with *Squalodelphis*, *Notocetus*, and Platanistidae. The fossils *Otekaikea marplesi, W. maerewhenua* and other waipatiid-like marine dolphins from New Zealand should help to understand the start of the radiation leading to the endangered Ganges river dolphin *Platanista gangetica*.

## Supporting Information

Appendix S1 **Cladistic matrix in nex format.**
(ZIP)

Appendix S2 **Cladistic matrix in TNT format.**
(ZIP)

Appendix S3 **Morphological characters used in the phylogenetic analysis.**
(DOCX)

Appendix S4 **List of coding modifications.**
(DOCX)
