# Supplementary material for: Fossil Dolphin Otekaikea marplesi (Latest Oligocene, New Zealand) Expands the Morphological and Taxonomic Diversity of Oligocene Cetaceans
Source: PLoS One. 2014 Sep 24;9(9):e107972. doi: 10.1371/journal.pone.0107972 (PMC4176723; doi:10.1371/journal.pone.0107972)
Supplement: Appendix S4 — List of coding modifications. (DOCX) [file pone.0107972.s004.docx]

Tanaka and Fordyce Appendix S4.

Appendix S4.

List of modifications to the original codings of Murakami et al. [[1](#_ENREF_1)] or the original codings of *Papahu taitapu* by Aguirre and Fordyce [[2](#_ENREF_2)]. The initial number refers to the character number in Appendix S3. Character numbers are identified by a hatch #. For some characters, a new state (generally 2) was added to indicate the hitherto uncoded situation in the outgroup taxon.

(2) Premaxillae transverse proportion: transversely inflated almost entire length of rostrum (0); flat almost entire length of the rostrum (1).

Character name and states now refer to a transverse orientation, to differentiate our premaxillary characters 2 and 3.

(3) Premaxillae mediolateral proportion: not compressed mediolaterally (0); compressed mediolaterally at anterior of rostrum (1).

Character name and states now refer to a mediolateral orientation, to differentiate our premaxillary characters 2 and 3.

(6) Mesorostral groove: V-shaped or U-shaped opening (0); partially or completely filled in by vomer to become a solid rod of bone (1); absent (2).

A new state, absent (2), for the mesorostral groove is added to indicate the state for Archaeoceti, and codings are changed thus:

*Georgiacetus vogtlensis* 0 to 2

*Zygorhiza kochii* 0 to 2

(7) Mesorostral groove constricted posteriorly, anterior to the nares and behind the level of the antorbital notch, then rapidly diverging anteriorly: absent (0); present (1).

“…constricted posteriorly, anterior to the nares and behind the level of the antorbital notch, then rapidly diverging anteriorly” is added after discussion with M. Murakami (personal communication) to clarify the original definition of the character.

*Georgiacetus vogtlensis* - to 0

*Zygorhiza kochii* - to 0

*Agorophius pygmaeus* 0 to ?, damaged.

(9) Rostral constriction: absent (0); constriction anterior to antorbital notch (1); constriction anterior to maxillary flange (2).

*Waipatia maerewhenua* 1 to 0

The rostral constriction is formed by intersection, in dorsal view, of the profiles of the maxillary flange and rostral outer margin in many Odontoceti. The more-dorsal maxillary flange runs obliquely anteriorly at an angle to the more-ventral rostral outer margin. *Waipatia maerewhenua* does not have the maxillary flange and lacks the rostral constriction.

(16) Posterior wall of antorbital notch: maxilla (0); lacrimal and jugal, or maxilla appeared in small area posterior to antorbital notch parallel with lacrimal and jugal (1); no notch but horizontal groove inferred to be for the facial nerve in the maxilla laterally on the face well above the margin of the rostrum (2).

The new state 2 is added for the condition in Archaeoceti and codings are changed as below.

*Georgiacetus vogtlensis* 0 to 2

*Zygorhiza kochii* 0 to 2

Character 19, in Murakami et al. [[1](#_ENREF_1)]. Large diastema between posterior cheek teeth: absent (0); present (1).

Deleted because variation in size of diastemata does not allow simple division into these two states. For example, most of the posterior diastemata in *Zygorhiza* are large in absolute length but small compared with tooth size. Likewise, *Platanista gangetica* shows different sizes of diastemata among posterior cheek teeth, from large anteriorly, to reduced or absent posteriorly. Size should be relative to a useful standard, with states redefined accordingly.

(21) Teeth: heterodont and some teeth with denticle (0); conical, with or without accessory cusp (1); spatulate (2); laterally compressed (3).

“Denticle” was changed from “dentine”.

(27) Anterior-most mandibular "tooth": about same size as posterior teeth (0); smaller than posterior teeth (1); greatly enlarged (2); forming a tusk (3).

*Waipatia maerewhenua* 0 to ?, because of damaged anterior end.

(29) Length of mandibular symphysis as percent of mandible length: long, >20% (0); short, <20% (1).

*Notocetus vanbenedeni* ? to 0

(30) Mandibular symphysis: sutured but unfused (0); fused (1).

*Notocetus vanbenedeni* 0 to 1

(31) Longitudinal groove on underside of mandibles: absent (0); present (1).

*Notocetus vanbenedeni* 0 to 1

(33) Elevation of coronoid process: very high (0); moderate (1); low (2).

Character name is changed from “coronoid crest” to “process” as Geisler and Sanders [[3](#_ENREF_3)] used originally. (The coronoid crest is the descending mandibular surface between the coronoid process and the tooth row.).

*Notocetus vanbenedeni* ? to 1

(34) Antorbital process of maxilla in dorsal view: triangular (0); robust and globose or rectilinear (1); absent (2).

The qualifier “of maxilla in dorsal view” is added in the character description.

A new state, absent (2), is added for Archaeoceti, and codings are changed as below.

*Georgiacetus vogtlensis* - to 2

*Zygorhiza kochii* - to 2

*Archaeodelphis patrius* 1 to 2, because the process lateral to the antorbital notch is formed by the lacrimall, not the maxilla as in other odontocetes. Thus, the process is not homologous in structure with the antorbital process.

*Xenorophus sloanii* 0 to 2, as for *Archaeodelphis patrius.*

(35) Angle of anterior edge of supraorbital process and the median line: anterior edge of supraorbital process: oriented slightly anterolaterally, at an angle <30° (±) with sagittal plane (0) oriented anteromedially (1).

The two states have been swopped, based on personal communication with with M. Murakami to clarify the original definition of the character.

(39) Lacrimal and jugal: separated (0); fused (1).

*Waipatia maerewhenua* ? to 1 (Aguirre and Fordyce [[2](#_ENREF_2)]; #53).

*Squaloziphius emlongi* 0 to 1

*Prosqualodon davidi* 0 to ?

*Platanista gangetica* ? to 1

(40) Lacrimal and jugal: contacting each other externally (0); lacrimal excluded from edge of skull, jugal directly contacting anterior edge of frontal (1).

*Waipatia maerewhenua* 0 and 1 to 0

(41) Jugal: thick and sturdy (0); thin, splint, or absent (1).

*Prosqualodon davidi* 1 to ?, because of damage.

(42) Combined anteroposterior length of the lacrimal and jugal exposure that is posterior to antorbital notch. With skull in ventral view, exposure is small and combined length forms <50% of anteroposterior distance from antorbital notch to postorbital ridge (0); intermediate, forms between 50 and 62% of that distance (1); large, forms between 62 and 69% that distance (2); very large, forms >69% of that distance (3).

*Notocetus vanbenedeni* ? to 1

*Prosqualodon davidis* 1 to 0 (Aguirre and Fordyce [[2](#_ENREF_2)]; #55).

*Pomatodelphis inaequalis* ? to -

(43) Dorsolateral edge of internal opening of infraorbital foramen: formed by maxilla (0); formed by maxilla and lacrimal and/or jugal (l); formed by lacrimal and/or jugal (2); formed by frontal (3).

*Waipatia maerewhenua* 1 to 1 and 3 (Aguirre and Fordyce [[2](#_ENREF_2)]; #57).

(45) Maxillary tuberosity: present (0); absent (1).

Character name is changed from “infraorbital plate” as applied to Archaeoceti *sensu* Geisler and Sanders [[3](#_ENREF_3)] to “maxillary tuberosity” of other mammals. Mead and Fordyce [[4](#_ENREF_4)] (infraorbital process) discussed names for the structure that protrudes posteriorly into the orbit.

Deleted state 2 from the original states, based on personal communication with with M. Murakami to clarify the character states.

(46) Direction of apex of postorbital process of frontal: projected posterolaterally and slightly ventrally (0); directed ventrally (1); not clear because of extremely reduced process (2).

State 2 applies to members of the Platanistidae which have extremely reduced postorbital processes without a clear orientation. For example, a *Platanista* skull with the rostrum level has a postorbital process oriented weakly posteriorly but, with the skull in living orientation (rostrum down, cranium up), the process orients ventrally. This condition might be related with having the thickened maxillary crests among the Platanistidae.

*Pomatodelphis inaequalis* 1 to 2

*Zarhachis flagellator* 0 to 2

*Platanista gangetica* 0 to 2

*Waipatia maerewhenua* 1 to 0

(47) Shape of postorbital process of frontal: robust, blunt descending posteriorly (0); pointed, attenuated, or acute triangular (1); triangular, trapezoidal, or an anteroposteriorly widened falciform (2); dorsoventrally long falciform (3).

State (0) “stick-like” is changed to “robust, blunt descending posteriorly”.

(51) Width of premaxillae at antorbital notches as percent width of rostrum at antorbital notch: narrow, <49% (0); moderate, 50–64% (1); wide, >65% (2); antorbital notch absent (3).

A state (3) “antorbital notch absent” is added for the Archaeoceti.

*Georgiacetus vogtlensis* - to 3.

*Zygorhiza kochii* - to 3.

(53) Size of premaxillary foramen: right and left subequal (0); right much larger than left (1); premaxillary foramen absent (2).

A state (2) “premaxillary foramen absent” is added for the Archaeoceti.

*Georgiacetus vogtlensis* - to 2.

*Zygorhiza kochii* - to 2.

(54) Position of premaxillary foramen: anterior of antorbital notch and anterior edge of supraorbital process (0); approximately medial to or posterior to antorbital notch region (1); premaxillary foramen absent (2).

A state (2) “premaxillary foramen absent” is added for the Archaeoceti.

*Georgiacetus vogtlensis* - to 2.

*Zygorhiza kochii* - to 2.

Character 56 in Murakami et al. [[1](#_ENREF_1)]. Premaxillae: contact both nasals (0); at least one not contacting nasals (1).

Deleted, because this character overlaps with #76 posterior projections of premaxillae.

(57) Posterolateral sulcus: deep (0); shallow or absent (1); presence of additional posterolateral sulcus (longitudinal striation) (2).

*Georgiacetus vogtlensis* - to 1, absent.

*Zygorhiza kochii* - to 1, absent.

(59) Posterior dorsal infraorbital foramina of maxilla: absent (0); one (1); two or more (2).

*Georgiacetus vogtlensis* - to 0

*Zygorhiza kochii* - to 0

Two characters in Murakami [[1](#_ENREF_1)]; #60 maxilla on dorsal surface of skull, and (62) anterolateral corner of maxilla overlying supraorbital process of frontal, are difficult to apply and code in some taxa. Here, the characters are changed back to the original characters used by Geisler and Sanders [[3](#_ENREF_3)] and Geisler et al., [[5](#_ENREF_5), [6](#_ENREF_6)] (character 62 and 64 in this study respectively).

(66) Maxillary crest on supraorbital process of maxilla: longitudinal ridges absent except at lateral edge of antorbital process (0); presence of longitudinal ridge except at lateral edge of antorbital process (1); longitudinal ridge present and joined with maxillary flange (2); presence of transversely compressed high crest, except at lateral edge of antorbital process (3); absent (4).

Character discription “maxillary ridge” was changed to “maxillary crest”.

State (4) is added for Archaeoceti.

*Georgiacetus vogtlensis* - to 4

*Zygorhiza kochii* - to 4

(68) Premaxillae in dorsal view: separated anterior to bony nares, exposing mesethmoid (0); joined premaxillae (or maxillae) closing at least posterior part of mesorostral groove (1).

“in dorsal view” was added.

(69) Anterior edge of bony nares: V-shaped, premaxillae gradually converging anteriorly to midline (0); U-shaped, premaxillae abruptly converging anteriorly to midline (1).

Character description now specifies “anterior edge of” the nares.

(71) Maxillary intrusion, anterior to external nares and encroaching the posteromedial or medial face of each premaxilla: absent (0);

maxilla visible within mesorostral groove as small exposure medially (1); exposure of maxilla reaches dorsally to level of premaxilla and forms a square or rectangular plate (2); exposure of maxilla reaches dorsally and forms a small subcircular to polygonal ossicle (3).

Character name is changed from “Medial maxilla-premaxilla suture or accessory ossicles” to "maxillary intrusion" of Arnold and Heinsohn [[7](#_ENREF_7)]. The original state of present is divided into three states. A distinct accessory ossicle is a synapomorphy of the Phocoenidae (Muizon [[8](#_ENREF_8)]).

(76) Ratio of greatest width of premaxillae to greatest width of maxillae at level of postorbital processes: ≥0.50 (0); 0.49–0.38 (1); <0.38 (2).

*Platanista gangetica* ? to 0

(77) Premaxillary eminence: absent (0); present but low (1); present and high (2).

*Georgiacetus vogtlensis* - to 0

*Zygorhiza kochii* - to 0

*Physeter catodon* - to 0

*Kogia breviceps* - to 0

(79) Left bony naris: same size or slightly larger than right bony naris (0); at least twice the size of right bony naris (1).

State (0) is changed from “same size as” to “same size or slightly larger than”.

(84) Inflections of ascending processes of premaxillae: gradual (0); vertical (1).

Character description was changed because the character originally referred to two different phenomena: the shape of the ascending process of the premaxilla, and the inclination of the premaxilla.

(85) Premaxillary cleft: absent (0); present, posterior part of ascending processes of premaxillae bearing a distinct cleft, originating at posterior edge of premaxillae and continuing anteriorly, dividing each premaxilla into two (1); present, with shallow cleft (2).

*Platanista gangetica* 0 to 2

*Prosqualodon davidis* 0 to ?

*Notocetus vanbenedeni* 0 to ?

*Zarhachis flagellator* 0 to 2

*Pomatodelphis inaequalis* 0 to ?

(94) Nasal-frontal suture: approximately straight transversely (0); anterior wedge (narial process) between frontal posterior ends of nasals (1); W or reversed U suture line (2).

“(narial process)” was added.

(97) Vertex: absent (0); present (1); highly developed (2).

Character name is changed from “synvertex”, because the term has been used for elevated vertex of the Ziphiidae (here, state 2 is equivalent to the synvertex).

(104) Parietal dorsally: not fused to frontal or supraoccipital (0); completely fused to, and indistinguishable from, frontal or supraoccipital (1).

"dorsally" and "other" were added.

(109) Zygomatic process of squamosal: directed anterolaterally (0); directed anteriorly (1).

*Papahu taitapu* 0 to 1. The left zygomatic process projects anterolaterally, judged to be the result of postmortem distortion,

(110) Zygomatic process of squamosal in lateral view: part of dorsal face visible (0); entire dorsal surface of squamosal visible (1).

The original inclusion of “sternomastoid fossa” is deleted, based on personal communication with with M. Murakami to clarify the character states.

(114) Postglenoid process of squamosal: not reduced (0); greatly reduced (1).

Postglenoid process is used in preference to “retroglenoid process”.

(115) Postglenoid process in lateral view: tapering ventrally (0); squared off ventrally (1); same as state 1 except very wide anteroposterior diameter of process (2).

Postglenoid process is used in preference to “retroglenoid process”.

*Papahu taitapu* 1 to –, ventral part of the postglenoid process and basioccipital crest have been eroded.

(116) Relative ventral projections of postglenoid and post-tympanic processes of squamosal: postglenoid process more ventral or at same level as post-tympanic process (0); apex of postglenoid process dorsally higher than post-tympanic process (1).

Postglenoid process is used in preference to “retroglenoid process”. State (1) “dorsally” is added.

*Waipatia maerewhenua* 1 to 0

(117) Nuchal crest in dorsoposterior view: semicircular, pointed anteriorly (0); rectangular or weakly convex anteriorly or posteriorly (1); convex posteriorly and/or midpoint convex triangular and pointed anteriorly (2); prominently convex anteriorly (3); strongly convex posteriorly (4).

Originally, state (4) was coded but was not in the character list. Now state (4) is added based on personal communication with with M. Murakami to clarify the character states.

(120) Palatine in nasal passage: thin, forming posterior part of nasal passage (0); thick, forming part of anterior wall of nasal passage (1); palatine does not join anterior wall of nasal passage (2).

In this character, “in nasal passage” is added to differentiate the character from (120) palatine exposure. State (2) was changed from "maxilla" to "palatine".

(121) Palatine exposure: exposed ventrally (0); partially covered by pterygoid, which divides it into medial and lateral exposures (1); ventral surfaces completely covered by pterygoids (2).

In this character, “exposure” is added to differentiate this character from (119) palatine in nasal passage.

(122) Lateral lamina of palatine: absent (0); present (1).

*Prosqualodon davidi* 1 to ?

(124) Lateral lamina of palatine: does not form bony bridge over orbit (0); does form bony bridge over orbit (1).

The misleading term "over" is qualified as ventral.

(133) Preorbital and postorbital fossae of pterygoid sinuses: widened apices of preorbital and postorbital fossae of pterygoid sinuses present but fossae not merged or fused (0); widened apices of preorbital and postorbital fossae of pterygoid sinuses merged or fused dorsal to path of optic nerve (1).

The term fossae is used instead of lobes.

(134) Fossa for preorbital lobe of pterygoid sinus in orbit: absent (0); present (1).

The fossa for the preorbital lobe of pterygoid sinus is in the orbit, not in the basicranium.

(138) Orbitosphenoid: not contacting lacrimal or lacrimojugal (0); contacting lacrimal or lacrimojugal (1).

Character terminology is changed from “lacrimal” to “lacrimal or lacrimojugal”

(139) Ratio of length of hamular process of pterygoid to cranium length: <0.30 (0); 0.30–0.44 (1); 0.45–0.59 (2); >0.60 (3).

"neurocranium" was changed to "cranium".

(142) Falciform process of squamosal: plate-like with anteroposteriorly wide base (0); rod-like with narrow base (1); poorly developed or absent (2).

*Papahu taitapu* - to 0 (Aguirre and Fordyce [[2](#_ENREF_2)]; #176).

(144) Tympanosquamosal recess: absent, with anterior transverse ridge present (0); anterior transverse ridge absent and middle sinus inferred to be present without a large tympanosquamosal recess (1); present and enlarged, forming triangular fossa medial and anteromedial to postglenoid process (2); very large, forming large fossa bordering entire medial edge of glenoid fossa (3).

Character state (1) is changed from absent to present, as recommended by personal comunication from Murakami. The loss of the anterior transverse ridge is a proxy for the presence of the middle sinus (Fordyce [[9](#_ENREF_9)]) which usually occupies a fossa, the tympanosquamosal recess. The original state “...small rectangular fossa for sigmoid process of tympanic bulla, present” in state (1) is also deleted, because the sigmoidal fossa (for the sigmoidal process of the bulla) is a separate character.

*Waipatia maerewhenua* 2 or 3 to 2

*Notocetus vanbenedeni* 3 to 2

(145) Bifurcation of tympanosquamosal recess: absent, almost undeveloped, elliptic (0); present, with a clear expansion anteriorly, invasion of mandibular fossa medially, and a depression (expansion) at the postglenoid process posteriorly (1).

“Pubic” is a meaningless description and is deleted from state 0. State 1 is expanded for clarification.

*Georgiacetus vogtlensis* 0 to -, because the character is irrelevant to Archaeoceti.

*Zygorhiza kochii* 0 to -, changed as for *Georgiacetus*.

(147) Position of more-distal part of alisphenoid-squamosal suture, with skull in ventral view: anterior to external opening of foramen oval or groove for mandibular nerve (0); courses along groove for mandibular nerve, or just posterior to it (1); just medial to anterior edge of floor of squamosal fossa, foramen ovale, and/or groove situated entirely on alisphenoid (2).

The term “groove for mandibular nerve” is used for clarity.

Character 148 in Murakami et al. [[1](#_ENREF_1)]. Periotic fossa: bowl-shaped (0); having transverse ridge, dividing periotic fossa into anterior and posterior portions (1); absent (2).

Deleted because of overlap with one character inolving the posterior portion of the periotic fossa (#151).

(149) Suprameatal pit of squamosal: absent (0); present but shallow, situated dorsolateral to spiny process of squamosal (1); forming deep dorsolateral excavation into squamosal (2).

*Waipatia maerewhenua* 2 to 1, because it is much shallower than *Zarhachis* which was coded as (2).

*Tursiops truncatus* 0 and 1 to 0, see Mead and Fordyce ([[4](#_ENREF_4)]: 107).

(151) Posterior portion of periotic fossa of squamosal: fossa absent (0); fossa present but shallow (1); highly compressed fossa forming narrow slit or small blind foramen (2); posteromedial portion contains large deep fossa (3).

*Waipatia maerewhenua* 0 or 1 to 1

*Papahu taitapu* - to 1 (#187; Aguirre and Fordyce [[2](#_ENREF_2)]).

(154) Vomer: posterior edge terminating on or at anterior edge of basisphenoid (0); terminating on basioccipital, covering basioccipital-basisphenoid suture ventrally (1).

*Waipatia maerewhenua* 1

(156) Posteroventral-most point of basioccipital crest: rounded over (0); forming closely appressed separate flange, with narrow crease separating it dorsally from rest of basioccipital crest (1); projecting distinct flange posteriorly (2); distinct but separated by pronounced notch, interrupting basioccipital crest (3).

*Physeter catodon* ? to 2

*Inia geoffrensis* 3 to 1 and 3. Inia has variation on the notch, which is the suture between the basioccipital and exoccipital. Some *Inia* specimens have a closed notch which looks like a crease (USNM 395614). The nature of ontogenetic variation is uncertain.

(157) Angle formed by basioccipital crests in ventral view: parallel with no angle formed (0); ca. 15–40° (1); ca. 42–68° (2); ca. 70–90° (3); >100° (4).

*Papahu taitapu* 2 to – (#194; Aguirre and Fordyce [[2](#_ENREF_2)]).

(158) Hypoglossal foramen: separated from jugular foramen, or jugular notch, by thick bone (0); separated by very thin bone or absent, in latter case hypoglossal foramen becoming confluent with jugular foramen (1).

*Papahu taitapu* - to 0 (#195; Aguirre and Fordyce [[2](#_ENREF_2)]).

(159) Jugular notch, gap between paroccipital process and basioccipital crest: open notch, width of opening and depth of notch about equal (0); narrow and almost slit-like, depth much greater than width of opening (1).

"Opening notch" was changed to "open notch".

(168) Articulation of anterior process of periotic to outer lip of tympanic bulla: contact of ventral surface of anterior process of periotic with outer lip of tympanic bulla (0); contact with thickened rim of outer lip of tympanic bulla and additionally with accessory ossicle (1); contact only with accessory ossicle (2).

Reference to processus tubarius was deleted. Note that the outer lip as used here is not the processus tubarius as cited by earlier users of the character; the latter is the accessory ossicle.

Character 169 in Murakami et al. [[1](#_ENREF_1)]. Lateral tuberosity of periotic separation from anterior process: by marked transverse groove (part of antero-external sulcus) in lateral view (0); absence of groove, lateral tuberosity continuous with anterior process (1).

Deleted; see below. New character (parabullary sulcus) substituted.

Character 170 in Murakami et al. [[1](#_ENREF_1)]. Lateral tuberosity of periotic in ventral view: short, narrow, and poorly individualized lateral tuberosity (0); tuberosity thickened, well developed laterally with an exposed ventral surface distinctly larger than mallear fossa (1).

Two lateral tuberosity characters (#169 and #170 in Murakami et al. [[1](#_ENREF_1)]) are deleted. The structure of the lateral tuberosity is highly influenced by development of the parabullary ridge, leading to some character overlap with parabullary ridge character (#170 in this study; #171 in Murakami et al. [[1](#_ENREF_1), [10](#_ENREF_10)]).

(169) Parabullary sulcus: absent (0); strongly curved, C-shape (1); weakly curved (2); strongly curved, V-shape (3) (modified from Fordyce, 1994 [56] Anteroexternal sulcus).

This new character is added; see text for discussion.

(170) Parabullary ridge of periotic: absent (0); present (1); present with a fossa between anterior process and parabullary ridge (2).

States are modified as discussed in the text. State (2) is seen in Ziphiidae, where a fossa forms a large triangular or crescentic depression between the anterior bullar facet and the lateral margin of the parabullary ridge, in contrast to the Delphinoidea in which the parabullary ridge passes medially toward the fovea epitubaria or, where it is present, the anterior bullar facet.

(171) Articulation of anterior process with squamosal: extensive, most of lateral side contacting squamosal (0); large centrally-oriented ovoid region contacting squamosal, free around edges (1); small area of contact with squamosal (2); contact absent, articulation via ligaments (3).

*Papahu taitapu* - to 0, (#207; Aguirre and Fordyce, [[2](#_ENREF_2)]).

(172) Anterior bullar facet: present (0); absent (1).

*Prosqualodon davidis* 1 to 0 (see Flynn [[11](#_ENREF_11)]; figure 6).

(173) Anterior incisure: deep, pocket-like fossa with anterior groove (0); anterior groove only (1).

Character name is changed (see Mead and Fordyce [[4](#_ENREF_4)]; 111).

(174) Fenestra rotunda: oval to subrounded (0); shaped like teardrop with fissure directed toward aperture for cochlear aqueduct (1).

Character description “perilymphatic foramen” is changed to “aperture for cochlear aqueduct”.

*Papahu taitapu* 0 to – (#222; Aguirre and Fordyce [[2](#_ENREF_2)]).

(175) Dorsal surface of periotic in lateral view: convex dorsally (0); pyramidal process convex dorsally (1); nearly flat (2).

*Notocetus vanbenedeni* 2 to 1

*Kentriodon pernix* 0 to 2

(176) Relative position of dorsal depth of stapedial muscle fossa and fenestra rotunda: ventral to, or in line with, dorsal edge of fenestra rotunda (0); well dorsal to fenestra rotunda (1).

Character description is changed from “posterodorsal edge of stapedial muscle fossa”.

(177) Posterodorsal edge of stapedial muscle fossa: absent, rounded lip (0); present (1).

Character state (0) is changed from “absent or reduced” to “absent, rounded lip”.

(178) Caudal tympanic process of periotic: low, its ventral and posterior edges drawing smooth curve (0); elevated, its ventral and posterior edges forming a right angle in medial view (1).

Character state (1) is changed from “prominent” to “elevated”.

*Mesoplodon ginkgodens* 1 to 0

*Tursiops truncatus* 0 to 1

(182) Fundus of internal acoustic meatus: funnel-like, smaller at blind end and wider near rim (0); tubular (1).

*Zarhachis flagellator* 0 to 1

*Pomatodelphis inaequalis* 0 to 1

(183) Internal acoustic meatus: pyriform (0); circular (1).

*Notocetus vanbenedeni* 1 to 0

(185) Aperture for vestibular aqueduct, in dorsal view: at transverse level of spiral cribriform tract (0); more lateral than spiral cribriform tract (1).

The term of “tractus spiralis foraminosus” is changed to “spiral cribriform tract”.

(186) Articular rim: absent (0); present but small, forming ridge anterolateral to articulation surface of posterior process of periotic and separated from it by sulcus (1); present, sigmoidal and laterally elongated with hook-like process (2).

In character state (1) “ridge fitting into corresponding cavity posterolateral and slightly dorsal to spiny process of squamosal” is deleted (see discussion of morphology).

(189) Angle between posterior process of periotic and long axis of pars cochlearis from dorsal or ventral views: >135° (0); ≤135° (1).

“From dorsal or ventral views” is added.

*Platanista gangetica* 0 to 1

(190) Facet for bulla on posterior process of periotic, parallel-sided; no (0); yes (1). (modified from Fordyce, 1994 #63).

This character is added.

(191) Ventral surface of posterior process of periotic, along a straight path perpendicular to its long axis: flat (0); concave (1); convex (2).

*Notocetus vanbenedeni* 0 to 1

(195) Anterior spine of tympanic bulla: absent (0); present but short (1); present and long (2).

*Waipatia maerewhenua* 0 to 1. An incipient anterior spine is present on *Waipatia*, which was mentioned by Geisler and Sanders [[3](#_ENREF_3)] as “does not contain a process that is clearly differentiated from the rest of the bulla”, and coded as absent. Here, we follow the incipient anterior spine is present (see Fordyce, 1994; figure 11, E). This is a part which is rarely preserved in fossils.

(198) Width of tympanic bulla as percentage of its length along its long axis: wide, ≥65% (0); narrow and long, ≤64% (1).

*Waipatia maerewhenua* 1 to 0 (72%).

(200) Lateral furrow of tympanic bulla: shallow groove (0); absent (1); deep, well-defined groove (2).

*Waipatia maerewhenua* 2 to 0

(202) Medial edge of sigmoid process: expanded anteriorly to appose lateral tuberosity of periotic (0); not articulating with squamosal or periotic (1).

Character description is changed from “dorsal edge of sigmoid process”, because dorsal or distal edge fits with the sigmoidal fossa on the squamosal.

(205) Size of posterior process of tympanic bulla: equal to or greater than total length of tympanic bulla (0); much smaller than total length of tympanic bulla (1).

The qualifier “total length” is added to the state description.

*Zygorhiza kochii* 0 to 1

(210) Dorsal margin of involucrum of tympanic bulla: not excavated (0); excavated just anterior to posterior process(1); excavated at mid-part of involucrum (2).

Character is clarified by use of “dorsal margin”. State 1 is qualified as “just anterior to posterior process”; state (2) is added.

The excavation of the involucrum varies. State (1) is seen in the Delphinoidea as a strong excavation just anterior to the posterior process, so that the dorsal surface of the involcurum has a sigmoidal outline in lateral view. State (2) is seen in the Ziphiidae, Kogiidae and Physeteridae as a relatively shallower excavation in the mid-length of the involucrum, followed by a posterior rise. Thus, from lateral view, the dorsal outline is more M-shaped than sigmoidal.

*Physeter catodon* 0 to 2

*Kogia breviceps* 0 to 2

*Tasmacetus shepherdi* 0 to 2

*Berardius bairdii* 0 to 2

*Ziphius cavirostris* 0 to 2

*Mesoplodon ginkgodens* 0 to 2

Character 211 in Murakami et al., [[1](#_ENREF_1)]. Involucrum: groove absent (0); bearing prominent transverse groove on dorsal surface, dividing involucrum into thicker posterior part and thinner anterior part (1).

Deleted because of overlap with another involucrum depression character (#210 in Murakami et al. [[1](#_ENREF_1)]).

(211) Ridge on inside of bulla: present, as transverse ridge extending laterally from involucrum and partially dividing cavum tympani into anterior and posterior portions (0); absent (1).

*Notocetus vanbenedeni* 1 to 0

(212) Ventromedial keel of tympanic bulla: present along entire length (0); terminating approximately at level of lateral furrow or mid-point of the tympanic bulla (1); poorly defined along entire length (2).

*Notocetus vanbenedeni* 2 to 0

*Kogia breviceps* 0 to 2

*Mesoplodon ginkgodens* 1 to 0

*Tasmacetus shepherdi* 1 to 0

(214) Basihyal and thyrohyal connection: unfused (0); fused (1).

“Connection” is added to discriminate from the next basihyal-thyrohyal character #216.

(215) Basihyal and thyrohyal shape: arched (0); angled (1).

“Shape” is added to discriminate from the previous basihyal-thyrohyal character #215.

(234) Anterior slope on scapula, between anterior angle and midpoint of glenoid fossa, with anterior and posterior margin of glenoid fossa on a plain: shallow, <35° (0); steeper, >35° (1).

These states and numbers are swapped as based on personal communication with with M. Murakami to clarify the character. Character description is changed from “anterior angle between scapula and midpoint of glenoid fossa”, to read “with anterior and posterior margin of glenoid fossa on a plane”.

(235) Posterior slope on scapula, between scapula and midpoint of glenoid fossa with anterior and posterior margin of glenoid fossa on a plain: shallow, <25° (0); steeper, >25° (1).

See previous character. Character description is changed from “anterior angle between scapula and midpoint of glenoid fossa” to read “with anterior and posterior margin of glenoid fossa on a plane”.

(236) Crest dividing between infraspinous fossa and teres fossa: weakly developed (0); strongly developed (1).

Character description is changed from “tears fossa” to “teres fossa”.

References

1. Murakami M, Shimada C, Hikida Y and Hirano H (2012) Two new extinct basal phocoenids (Cetacea, Odontoceti, Delphinoidea), from the upper Miocene Koetoi Formation of Japan and their phylogenetic significance. J Vertebr Paleontol 32: 1172-1185.
2. Aguirre-Fernández G and Fordyce RE (2014) *Papahu taitapu*, gen. et sp. nov., an early Miocene stem odontocete (Cetacea) from New Zealand. J Vertebr Paleontol 34: 195-210.
3. Geisler JH and Sanders AE (2003) Morphological evidence for the phylogeny of Cetacea. J Mamm Evol 10: 23-129.
4. Mead JG and Fordyce RE (2009) The therian skull: a lexicon with emphasis on the odontocetes. Smithson Contrib Zool 627: 1-248.
5. Geisler JH, McGowen MR, Yang G and Gatesy J (2011) A supermatrix analysis of genomic, morphological, and paleontological data from crown Cetacea. BMC Evol Biol 11: 112.
6. Geisler JH, Godfrey SJ and Lambert O (2012) A new genus and species of late Miocene inioid (Cetacea, Odontoceti) from the Meherrin River, North Carolina, USA. J Vertebr Paleontol 32: 198-211.
7. Arnold PW and Heinsohn GE (1996) Phylogenetic status of the Irrawaddy dolphin *Orcaella brevirostris* (Owen in Gray): a cladistic analysis. Mem Queensland Mus 39: 141-204.
8. de Muizon C (1988) Les relations phylogenetiques des Delphinida (Cetacea, mammalia). Ann Paleontol 74: 159-227.
9. Fordyce RE (2002) *Simocetus rayi* (Odontoceti: Simocetidae, new family): A bizarre new archaic Oligocene dolphin from the eastern North Pacific. Smithson Contrib Paleobiol 93: 185-222.
10. Murakami M, Shimada C, Hikida Y and Hirano H (2012) A new basal Porpoise, *Pterophocaena nishinoi* (Cetacea, Odontoceti, Delphinoidea), from the upper Miocene of Japan and its phylogenetic relationships. J Vertebr Paleontol 32: 1157-1171.
11. Flynn TT (1948) Description of *Prosqualodon davidi* Flynn: a fossil cetacean from Tasmania. 26: 153-197.
